# Effects of *Nervilia fordii* Extract on Pulmonary Fibrosis Through TGF-β/Smad Signaling Pathway

**DOI:** 10.3389/fphar.2021.659627

**Published:** 2021-04-19

**Authors:** Yufeng Yao, Yue Yuan, Zenghui Lu, Yunxia Ma, Yuanyuan Xie, Meiqi Wang, Fangle Liu, Chenchen Zhu, Chaozhan Lin

**Affiliations:** ^1^ School of Pharmaceutical Sciences, Guangzhou University of Chinese Medicine, Guangzhou, China; ^2^ School of Basic Medical Sciences, Guangzhou University of Chinese Medicine, Guangzhou, China

**Keywords:** pulmonary fibrosis, *Nervilia fordii*, TGF-β/Smad signaling pathway, Network pharmacology analysis, effective substances and mechanism

## Abstract

Idiopathic pulmonary fibrosis (IPF) is a progressive and irreversible interstitial pulmonary disease with a poor prognosis. The extract of *Nervilia fordii* (NFE) has shown remarkable benefit in the treatment of acute lung injury, lung cancer, and severe acute respiratory syndrome (SARS). However, the potential mechanism and efficacy of NFE in the treatment of IPF remain unknown. In this study, a systematic network pharmacology analysis was used to predict the mechanism and efficacy of NFE in the treatment of IPF, based on the major components of NFE elucidated by UPLC-TOF-MS/MS. The potential molecular interactions between the compounds and potential targets were predicted using molecular docking. *In vivo*, rats with pulmonary fibrosis induced by a single intratracheal injection of bleomycin (BLM) were orally administered NFE for 14 days. Lung index and biochemical levels were determined, and histopathological analysis using hematoxylin and eosin (H&E) and Masson staining was performed. The effects of NFE on fibroblast proliferation in Lipopolysaccharide (LPS) and TGF-β1-induced mouse 3T6 fibroblasts were evaluated *in vitro*. In total, 20 components were identified in NFE, and 102 potential targets for IPF treatment were predicted. These targets potentially participate in processes regulated by transmembrane receptor protein tyrosine kinase, ERBB2, and et al. Molecular docking results predicted high affinity interactions between three components (rhamnazin, rhamnetin, and rhamnocitrin) and the potential targets, suggesting that TGF-β is the most important potential target of NFE in the treatment of pulmonary fibrosis. NFE significantly decreased the lung index and alleviated BLM-induced pulmonary fibrosis in rats. Histopathological observation of lung tissues showed that NFE alleviated inflammation and collagen deposition in BLM-induced rats. NFE inhibited the migration of LPS- and TGF-β1-induced 3T6 fibroblasts, reduced the contents of hydroxyproline and collagen, and contributed to anti-inflammation and anti-oxidation. With the intervention of NFE, the protein and RNA expression of TGF-β1, *a*-SMA, Smad3/4, *p*-Smad3/4, CTGF, and *p*-ERK1/2 were significantly downregulated, while Smad7 and ERK1/2 were upregulated significantly *in vivo* and *in vitro*. These findings indicated that NFE may exert therapeutic effects on pulmonary fibrosis by alleviating inflammation, oxidation, and collagen deposition. The mechanism related to the inhibition of the TGF-β/Smad signaling pathway.

## Introduction

Idiopathic pulmonary fibrosis (IPF) is a life-threatening disease characterized by a chronic inflammatory response, excessive proliferation of fibroblasts, aberrant deposition of extracellular matrix (ECM), and abnormal repair and remodeling of lung tissue (Wilson and Wynn, 2009; Sgalla et al., 2018). Studies have shown that several factors, such as environmental pollution, smoking, bacterial, and viral infection, including SARS-CoV and MERS-CoV, can lead to severe lung inflammation, poor wound healing response, permanent organ damage, and fibrosis. Similarly, there is emerging evidence that SARS-CoV-2, the virus that causes Coronavirus disease 2019 (COVID-19) can also induce fibrotic damage to infected tissues, and this complication can persist beyond the period of viral infection (George et al., 2020; Ojo et al., 2020). IPF patients are characterized by irreversible destruction of lung architecture associated with a progressive accumulation of fibrosis, and their prognosis is poor, with a median survival of less than 3 years (Wells and Rosas, 2016). The combination of glucocorticoids (such as prednisone), azathioprine, and N-acetylcysteine is the conventional treatment for patients with IPF, which can inhibit inflammation, suppress the immune response, and alleviate signs and symptoms of the disease. Pirfenidone and nintedanib are two newly developed anti-pulmonary fibrosis drugs that significantly improve function and delay the progression of IPF (Brunnemer et al., 2018; Yoon et al., 2019; Chung et al., 2020). However, owing to their high price and side effects, the use of these drugs is often limited in clinical settings. Because effective conventional drugs against IPF are limited, new therapeutic drugs and approaches are needed.

Herba seu Rhizoma Nervilia Fordii, dried leaves or leaves with bulbs of *Nervilia fordii* (Hance) Schltr. (family Orchidaceae), is a Chinese folk medicine that has been commonly used for the treatment of lung diseases, such as cough, pharyngitis, *tuberculosis*, and pneumonia for many years (Xu et al., 2010; Xie et al., 2018). Pharmacological studies have shown that *N. fordii* can inhibit lung cancer and acute lung injury by eliminating pulmonary edema and alleviating lung damage (Zhen et al., 2007; Xu et al., 2010; Lin et al., 2020). Phytochemical studies on *N. fordii* have resulted in the isolation of a series of flavonoids, phenolic acids, sterols, and triterpenoids, in which flavonoids are considered the main characteristic constituents (Lu et al., 2009; Tian et al., 2009; Zhang et al., 2011; Zhang et al., 2012; Chen et al., 2013; Qiu et al., 2013; Liu et al., 2015). Moreover, it has been reported that the flavonoids from *N. fordii* have potent anti-inflammatory and protective effects against acute lung injury, and have therapeutic effects in SARS (Pan et al., 2003; Zhen et al., 2007; Xu et al., 2010; Lin et al., 2020). However, the potential anti-fibrotic mechanism of *N. fordii* in pulmonary fibrosis is yet to be elucidated.

Injury of alveolar epithelial cells, activation of inflammation, and epithelial-mesenchymal transition (EMT) are typical characteristics of IPF development. Transforming growth factor-β (TGF-β) plays a central role in the promotion of lung fibrosis (Willis and Borok, 2007; Jung et al., 2012). TGF-β induces differentiation of lung fibroblasts into myofibroblasts, expression of *α*-smooth muscle actin (*α*-SMA), and synthesis of collagen to form ECM. With the activation of TGF-β, Smad3/4 is recruited and combined with TGF-β to form a complex that translocates from the cytoplasm into the nucleus, leading to the transcription of fibrosis-related genes, such as *α*-SMA and Col-I. Additionally, many studies have confirmed that connective tissue growth factor (CTGF) plays the role of a modulator of fibrosis progression induced by TGF-β. Dysregulation of CTGF has been linked to various fibrotic processes including the growth of fibroblasts and the secretion of ECM (Wuyts et al., 2013; Park et al., 2016), and this biological function was widespread in various fibrotic diseases, such as lung (Tzortzaki et al., 2007), liver (Arauz et al., 2013; Zhou et al., 2015; Han et al., 2019), kidney (Mao et al., 2020) and dermal fibrosis (Nakai et al., 2019). Among the CTGF signaling implicated in TGF-β responses, aberrant activation of the extracellular signal-regulated kinase (ERK) occurs and is phosphorylated to *p*-ERK (Kato et al., 2014; Chang et al., 2015), which further stimulates proliferation and collagen synthesis. Thus, TGF-β/Smad may be key therapeutic targets for IPF and fibroblast activation. Inhibition of TGF-β can significantly improve airway and lung static compliance, reduce collagen accumulation, and downregulate the expression levels of inflammatory factors (Cheresh et al., 2013; Lucarini et al., 2017; Sun et al., 2018; Liu et al., 2020).

In this study, a network pharmacology approach based on the components of *N. fordii* extract (NFE) and molecular docking methods were used to predict the effective components and therapeutic targets of NFE. Moreover, lipopolysaccharide (LPS), TGF-β1 and bleomycin (BLM), the chemicals that can cause pulmonary fibrosis (Brass et al., 2008), were used to induce IPF in a swiss-3T6 cell model and an animal model in this experiment, respectively. The effects and potential mechanisms of NFE on pulmonary fibrosis were investigated in BLM or LPS/TGF-β1 -induced pulmonary fibrosis *in vivo* and *in vitro*.

## Materials and Methods

### Chemicals and Reagents

BLM was manufactured by Nippon Kayaku Co., Ltd (batch number: 640412). Prednisone acetate was purchased from Guangdong South China Pharmaceutical Group Co., Ltd (batch number: 140704). Lipopolysaccharide (LPS) was obtained from Sigma-Aldrich (St. Louis, MO, United States, batch number: 025M4040Y). Recombinant mouse TGF-β1 was purchased from PeproTech (No. 218, Xinghu St, SIP Suzhuo, China) and used at a concentration of 5 ng/ml. N-acetylcysteine was produced by MedChemExpress LLC (Princeton, NJ, United States of America, batch number: HY-B0215/CS-2160). Primary antibodies against TGF-β1, *α*-SMA, Smad3, Smad4, *p*-Smad3, *p*-Smad4, Smad7, CTGF, ERK1/2, *p*-ERK1/2, *β*-actin, and GAPDH were purchased from Abcam (Cambridge, United Kingdom).

### Preparation of NFE

Dried whole plants of *Nervilia fordii* (Hance) Schltr. were collected from Guangdong Province, China, and authenticated by Prof. Danyan Zhang, Department of Pharmacognosy, Guangzhou University of Chinese Medicine. The voucher specimen (No. QTK-201701) was deposited at the Department of Phytochemistry, Guangzhou University of Chinese Medicine.

The *N. fordii* extract (NFE) was prepared using the following methods: The dried powder of *N. fordii* was refluxed with 80% aqueous ethanol (1:10, w/v) twice for 1 h, after which the filtrate was collected and concentrated. The filtrate was adjusted to 2.0 mg/ml (w/v) and purified using the AB-8 macroporous resin with gradient elution of ethanol; the 70% ethanol fraction was collected and freeze-dried to obtain the extract of *N. fordii* (NFE).

The quality of the NFE sample was controlled using qualitative analysis and the contents of the main components. The composition of NFE was analyzed using LC-Q-TOF-MS/MS, and 20 compounds were identified. The contents of five main components determined by UPLC/MS were 42.59 mg/g of rhamnocitrin 3,4′-O-glucoside, 13.12 mg/g of nervilifordin D, 0.79 mg/g of nervilifordin B, 0.25 mg/g of rhamnazin 3-O-glucopyraconoside, and 0.38 mg/g of rhamnocitrin. (Details are shown in Supplementary Material Part A).

### Network Pharmacology Prediction of the Potential Targets of Flavonoids in NFE

#### Screening Therapeutic Targets of Flavonoids in NFE Related to IPF

The targets of 20 NFE compounds were obtained from TCMSP (http://lsp.nwsuaf.edu.cn/tcmsp.php), STITCH (https://stitch.embl.de/), and SwissTargetPrediction (http://www.swisstargetprediction.ch/). The UniProt database (http://www.uniprot.org/) was used to standardize the target gene names, and duplicate targets were removed. The obtained targets were further filtered according to their relevance to IPF. “Pulmonary fibrosis,” “idiopathic pulmonary fibrosis”, and “IPF” were inputted as keywords in databases, including OMIM (https://www.ncbi.nlm.nih.gov/omim/), PubMed (https://www.ncbi.nlm.nih.gov/pubmed/), and Genecards (https://www.genecards.org/), to search for disease targets. Finally, the targets of flavonoids were mapped to the targets of the disease to identify therapeutic targets of NFE for the treatment of IPF.

#### Network Construction and the Target Pathway Analysis

To establish a relationship between compounds, targets, and diseases, Cytoscape 3.2.1 (http://www.cytoscape.org/) was used to construct the networks, including compound-compound target (C-C) network and compound-therapeutic target (C-T) network. The protein-protein interaction (PPI) network construction was analyzed using the STRING database (http://string-db.org/) and Cytoscape software. Furthermore, the potential effect of NFE on IPF was revealed by KEGG Pathway enrichment using the DAVID (https://david.ncifcrf.gov/) database.

#### Molecular Docking

The structural information (2D and 3D) of the compounds was obtained from the PubChem database (https://pubchem.ncbi.nlm.nih.gov/). The molecular structure was constructed using ChemDraw and Chem3D software (ChemOffice 17.0). The protein crystal structure was acquired from the RCSB Protein Data Bank (https://www.rcsb.org/) in PDB format. Before molecular docking, redundant sequences, water, ligands, and sequences were removed from the protein crystal structure. Molecular docking was performed using Discovery Studio 2.5 software. The chemical structures of the compounds were set as ligands and parameterized with the CHARMM General Force Field. The implement of molecular docking analysis and visualization were carried out using Discovery Studio Visualizer.

### Animals, Grouping, and Experimental Design

Fifty-six male Sprague-Dawley rats (200–250 g) were obtained from the Laboratory Animal Center of Guangzhou University of Chinese Medicine (Guangzhou, China, license: SYXK (Yue) 2018–0,001). All animals were housed in a controlled environment at room temperature (22 ± 2°C) and humidity (55 ± 5%), and had ad libitum access to food and water. Animal welfare and experimental procedures were strictly in accordance with the guidelines approved by the Animal Ethics Committee of Guangzhou University of Chinese Medicine.

The dosage of NFE was determined based on the results of an acute toxicity test, as shown in the supplementary material (Part C). After 1 week of acclimatized feeding, fifty-six rats were randomly divided into seven groups of eight rats each as follows: control group, sham group, model group, positive group (prednisone acetate, 3.5 mg/kg, i.g.), low-dose NFE group (100 mg/kg, i.g.), medium-dose NFE group (200 mg/kg, i.g.), and high-dose NFE group (400 mg/kg, i.g.). After anesthetization with sodium pentobarbital (6 mg/100 g body weight, i.p.), the BLM-induced rats were administered a single intratracheal instillation of BLM dissolved in physiological saline (4 mg/kg body weight), and the rats in the sham group were administered the same volume of physiological saline. 1 day after the operation, all drug treatments were administered for 14 consecutive days, while equal volumes of physiological saline were administered to the rats in the control, sham, and model groups. All rats were monitored daily to record their body weights.

Two hours after the final treatment, all rats were anesthetized with sodium pentobarbital (6 mg/100 g body weight, i.p.) before collecting blood samples via the abdominal aorta. The blood samples were centrifuged at 1500 ×g for 10 min at 4°C to obtain the serum, which was stored at −80°C for subsequent experiments. Lung tissues were excised from each rat, washed in ice-cold saline, dried with filter paper, and weighed. Lung tissues were stored at −80°C and fixed with 10% formalin. The lung index (LI) was calculated as follows: LI = lung weight (mg)/body weight (g).

### Histopathological Observation

The lungs were fixed, paraffin-embedded, sectioned at 4 μm, and stained with hematoxylin and eosine (H&E) (Solarbio, Beijing, China) for microscopic examination of morphological changes. The sections were also subjected to Masson staining (Solarbio, Beijing, China) to identify collagen fibers. Briefly, the sections were incubated with hematoxylin for 8 min to stain the nuclei and then with Ponceau-Fuchsin acid for 8 min to stain the cytoplasm. After washing with 0.2% glacial acetic acid, the sections were incubated with 1% phosphomolybdic acid for 5 min to destain the connective tissue, and the collagen fibers were stained with aniline blue (2% w/v aniline blue +2% v/v glacial acetic acid) for 1 min. The sections were observed using a microscope (Olympus, Shinjuku, Japan).

### Cell Culture and Treatment

The murine fibroblast cell line 3T6-Swiss albino (3T6) was purchased from the Cell Bank of the China Academy of Sciences (Shanghai, China). The cells were cultured in DMEM (Gibco, Carlsbad, CA, United States) supplemented with 10% fetal bovine serum (FBS; Gibco, Carlsbad, CA, United States) at 37°C in a humidified atmosphere of 95% air and 5% CO_2_. The 3T6 cells were stimulated with LPS (10 μg/ml) and treated with NFE (500, 200, and 100 μg/ml) or N-acetylcysteine (NAC, a positive drug) for 24 h. Meanwhile, 3T6 cells treated with an equal amount of PBS or NFE (500 μg/ml) were used as the control group and NFE-treated control group, respectively.

### Scratch Assay

Cell migration was assessed using a well-established *in vitro* scratch wound assay (Liang et al., 2007). A scratch was evenly created by horizontally crossing the surface of the cell monolayer with a 200 μL pipette tip. The exfoliated cells were washed off with a serum-free medium, and the adherent cells were cultured with 10% FBS (DMEM medium), which contained LPS, NFE, or NAC, for 24 h. The scratch-prefixed points were selected for capturing representative images (4 replicates per group) using a phase-contrast microscope (Olympus, Tokyo, Japan) at 0, 3, 6, 9, 12, and 24 h after scratching, and the blank areas were measured by Image-pro Plus 6.0 software (Media Cybernetics, Maryland, MD, United States). The percentage of wound closure was calculated as follows: Cell migration rate (%) = (Area _0h_−Area _indicated time point_)/Area _0h_ × 100%.

### Biochemical and Cytokine Analysis

The contents of hydroxyproline (HYP), myeloperoxidase (MPO), total antioxidant capacity (T-AOC), glutathione (GSH), and superoxide dismutase (SOD) in serum, lung tissue, and cell supernatant were determined using commercial kits according to the manufacturer’s instructions. The levels of collagen-1 (Col-1), collagen-3 (Col-3), tumor necrosis factor-*α* (TNF-*α*), and interleukin-1β (IL-1β) were measured using ELISA kits according to the manufacturer’s protocol.

### Western Blotting

The homogenate of lung tissue or 3T6 cell lysate was lysed in RIPA lysis buffer (Biyuntian, China) containing a blended protease inhibitor to obtain total proteins. Protein concentrations of the samples were measured using a BCA assay kit. Equal amounts of protein (30 μg each sample) were separated by 10% SDS-PAGE and transferred to 0.45 μm PVDF membranes (Millipore, Billerica, United States) and blocked with 5% BSA or 5% skimmed milk for 2 h before incubation with primary antibodies against TGF-β1 (1:1,000), *α*-SMA (1:1,000), Smad3 (1:3,000), Smad4 (1:3,000), *p*-Smad3 (1:1,000), *p*-Smad4 (1:2,000), Smad7 (1:1,000), CTGF (1:1,000), ERK1/2 (1:2,000), *p*-ERK1/2 (1:2,000), *ß*-actin (1:3,000), and GAPDH (1:3,000) for 14 h at 4°C. After washing with TBST, the membranes were incubated with HRP-labeled goat anti-rabbit IgG (1:5,000) for 1 h at room temperature, and the bound antibody was detected using an enhanced chemiluminescence detection reagent (Bio-Rad, California, CA, United States). Protein expression was quantified using densitometric analysis of three independent blots collected by the Bio-Rad ChemiDoc XRS + chemiluminescence imaging system (Bio-Rad, Hercules, CA, United States). *β*-actin and GAPDH were used as the loading controls.

### qRT-PCR Analysis

Total RNA was extracted from lung tissues using TRIzol reagent (Invitrogen^™^, Thermo Fisher Scientific, United States) according to the manufacturer’s protocol. The purity and concentration of total RNA were determined using a Nanodrop 4,000 spectrophotometer (Thermo Fisher Scientific, MA, United States). Reverse transcription and real-time PCR were performed using a Bestar® one-step RT qPCR kit (DBI Bioscience, Ludwigshafen, Germany). All reactions were performed in triplicate and data were analyzed according to the 2^−ΔΔCt^ method. The primer sequences used are summarized in the supplementary material (Part D).

### Statistical Analysis

Data are expressed as mean ± SEM of at least three independent experiments. The student’s t-test was used for statistical analysis using SPSS (version 19.0, Chicago, IL, United States). A *p* < 0.05, indicated a statistically significant difference, whereas a *p* < 0.01, indicated a significant difference.

## Results

### Network Pharmacology and Molecular Docking Study

**FIGURE 1:**
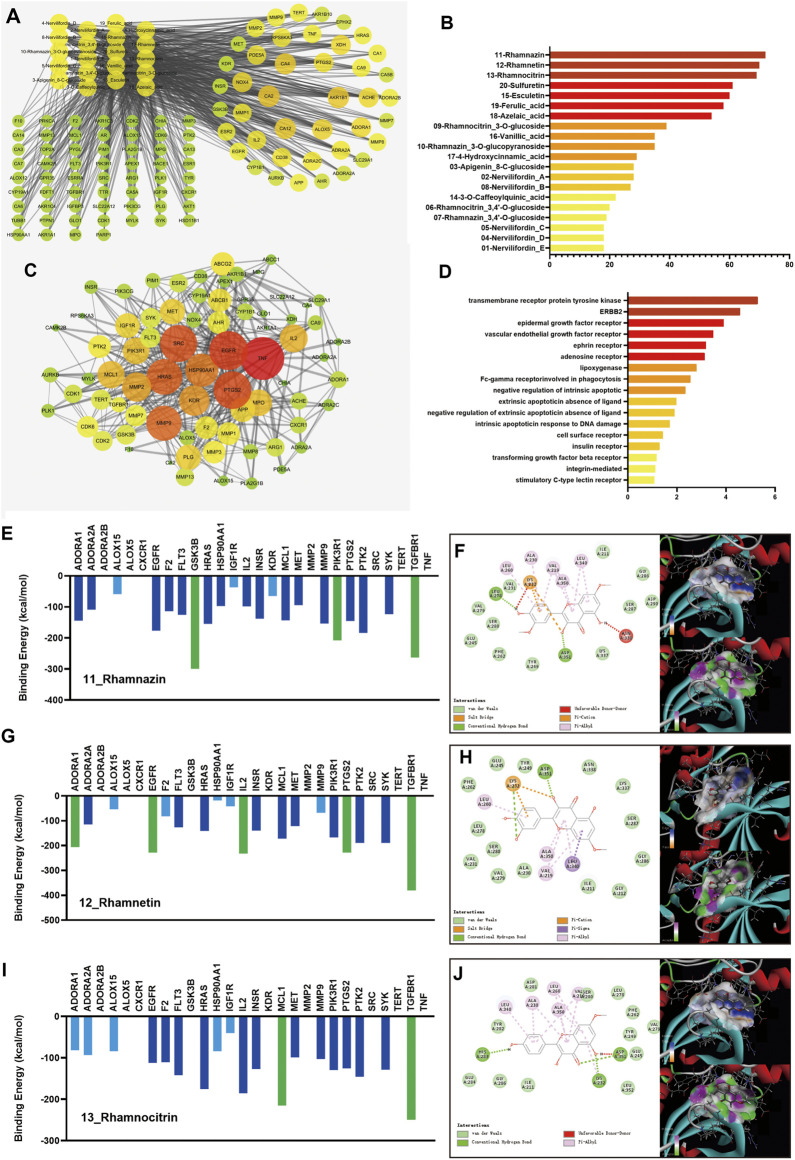
Results of the network pharmacology research and molecular docking. The component-target network of 20 active components and 102 targets of NFE were constructed **(A)**, and the degrees of 3 flavonoids (rhamnazin, rhamnetin, and rhamnocitrin) were higher than the others **(B)**. The targets were selected for the protein-protein interaction (PPI) network **(C)**. The pathways were re-analyzed by DAVID **(D)**. The docking results of rhamnazin, rhamnetin, and rhamnocitrin are shown **(E,G,I)**, with the 2D and 3D position of the ligand and the receptor **(F,H,J)**.

The components of NFE were analyzed using UPLC-Q-TOF-MS in both ESI+ and ESI− modes. A total of 20 compounds were qualitatively identified (Supplementary Materials Supplementary Figure S2) in light of the typical fragment ions and characteristic structure, which were considered as potential components in network pharmacology research. Using databases and structure-based target prediction, 604 potential gene targets were obtained corresponding to the components of NFE. A total of 5148 pulmonary fibrosis-related targets were identified after searching the disease databases. For the prediction of key compounds and targets for fibrosis treatment by NFE, a component-target (C-T) network (Figure 1A) was performed based on target mapping between compound targets and pulmonary fibrosis-related targets, and 102 targets with a C-T network degree greater than 3 were integrated and identified as potential therapeutic targets of NFE. Among the 20 ingredients, three flavonoids had higher target degrees (N) than the other candidates, including rhamnazin (*n* = 72), rhamnetin (*n* = 70), and rhamnocitrin (*n* = 69), which were indicated to have components for the most probable therapeutic effect (Figure 1B).

Considering that the important and influencing factors of these targets are not the same, the results of C-T network dose not fully reveal and explain the possible effects and mechanisms of candidate compounds in NFE. Potential therapeutic targets were selected and imported into the STRING database, and a PPI network was constructed (Figure 1C). To further extract the key potential interacting proteins from the whole PPI network, the top 50 targets were chosen and re-analyzed using DAVID functional annotation, which revealed significant changes in the pathways regulated by transmembrane receptor protein tyrosine kinase, ERBB2, epidermal growth factor receptor, and other pathways (Figure 1D). The important targets involved in these pathways may be related to the biological activity of the compounds in NFE, and those protein receptors have been selected to predicted the potential mechanism of the flavonoids on the treatment of pulmonary fibrosis.

To predict the activities of the three flavonoids on the targets, a protein-ligand docking simulation was performed. As shown in Figure 1E,G,I, the values of molecular docking binding energy of every molecule/ligand and protein/receptor were displayed, and the relative binding affinity was determined based on the binding energy. The results indicated that several protein receptors, including GSK3, PIK3R1, TGFBR1, ADORA1, EGFR, IL2, PTGS2, and MCL maintained effective docking activities with three flavonoids. The binding energy of these targets was less than −200 kcal/mol, marked as green in the figure. TGFBR1 has high docking activity for all three flavonoids. To gain further insight, we carefully checked the 2D and 3D positions of the ligand and receptor in the docking results and strikingly, the docking between the ligand and the receptor could be expressed in terms of different energy sources including electrostatic energy, Van der Waals energy, hydrogen energy, and so on, as shown in Figure 1F,H,J. It can be predicted that TGFBR1 and TGF-β signaling pathways are more likely to be the main mechanisms of NFE.

### NFE Alleviated BLM-Induced Pulmonary Fibrosis in Rats

**FIGURE 2:**
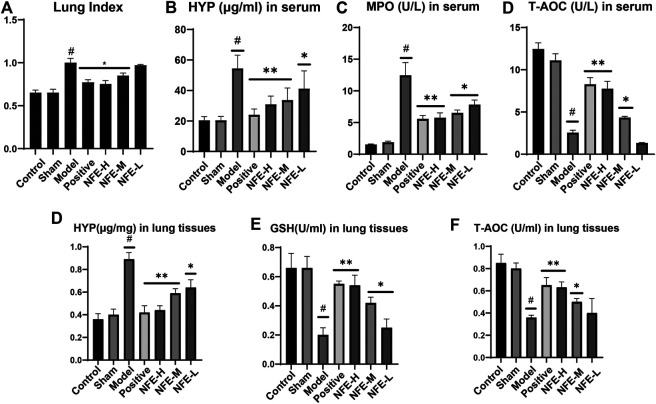
NFE alleviated BLM-induced pulmonary fibrosis in rats. Effects of NFE on lung/body weight ratio (Lung index, LI) of rats **(A)**. Effects on the contents of hydroxyproline (HYP) **(B)**, myeloperoxidase (MPO) **(C)**, and total antioxidant capacity (T-AOC) **(D)** in serum and effects on the contents of HYP **(E)**, glutathione (GSH) **(F)**, and T-AOC(G) in lung tissues. #*p* < 0.01 compared with Control group. **p < 0.01 compared with Model group. *p < 0.05 compared with Model group.

As shown in Figure 2A, BLM instillation led to a significant increase of LI compared with normal control rats. However, the increased LI in BLM-instilled rats was significantly reduced by NFE treatment.

The effect of NFE on HYP in serum and lung tissues of rats, model rats, and normal control rats is shown in Figure 2B–F. NFE (400 mg/kg and 200 mg/kg) treatment normalized the serum biomarker activities in a dose-dependent manner. T-AOC, GSH, and MPO are commonly used biochemical markers to express body oxidation and antioxidant imbalances. The levels of MPO and T-AOC in serum, GSH, and T-AOC in the lung tissue of rats were determined. A significant increase in MPO was observed in BLM-induced fibrotic rats when compared with normal control rats, while GSH activity was reduced. NFE (400, 200, and 100 mg/kg) treatment significantly restored the levels of MPO and GSH in a dose-dependent manner.

### Effect of NFE on Lung Histology in Rats

**FIGURE 3:**
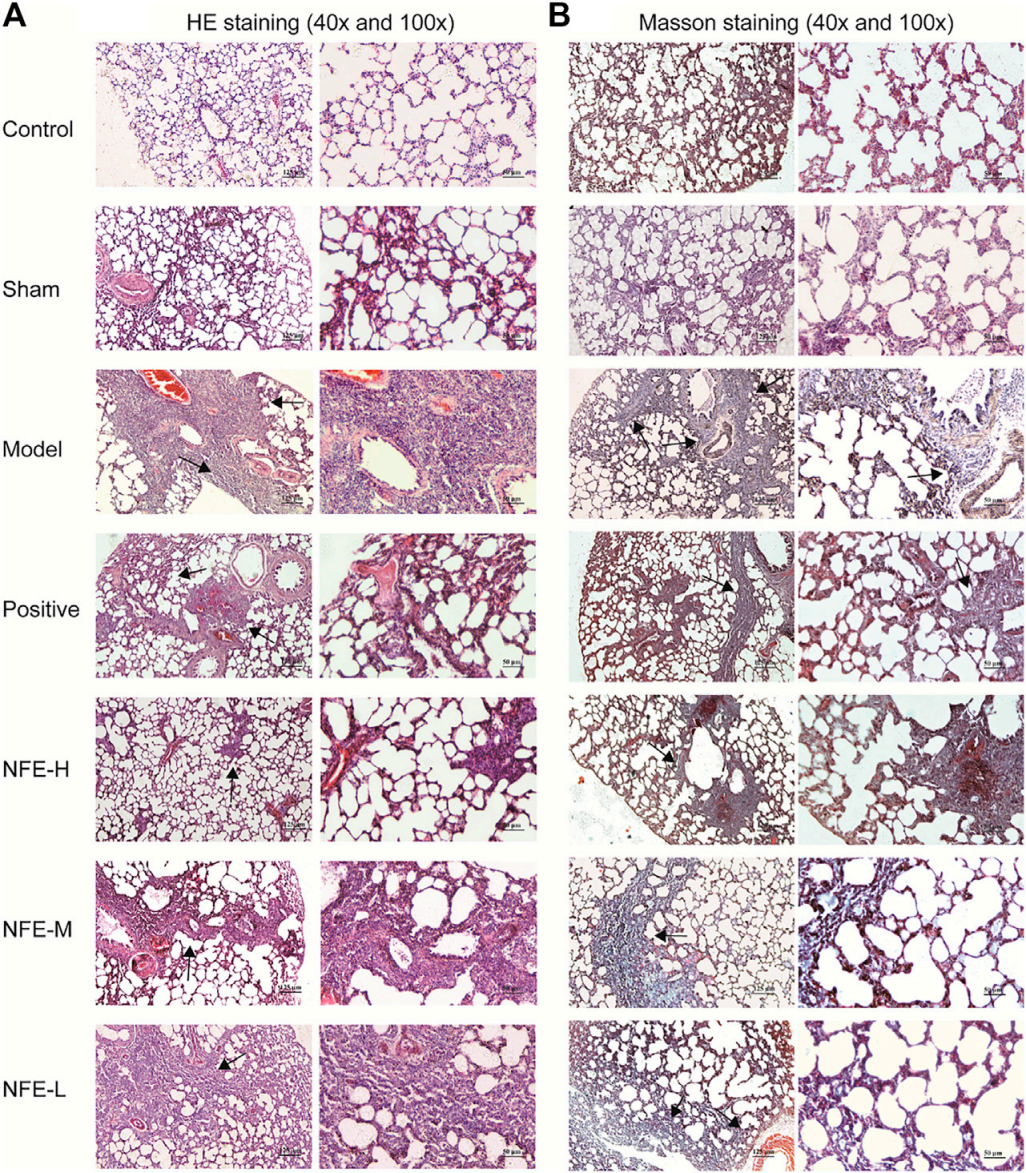
HE staining **(A)** and Masson staining **(B)** of lung tissue among different groups of SD rats (40x and 100x).

HE staining of lung sections of rats administered BLM for 14 days showed abundant inflammatory cells, fibrotic changes, and proliferation of granulation tissue. Lung sections of rats treated with NFE (400, 200, and 100 mg/kg) showed less fibrotic changes and inflammatory relief than those in the model group (Figure 3A).

Masson's trichrome staining of lung sections with BLM induced an abundance of collagen fibers when compared with normal control rats, while NFE (400, 200, and 100 mg/kg) treatment significantly reduced collagen deposition (Figure 3B).

### Effect of NFE by Regulating the TGF-β/Smad Pathway in Rats

**FIGURE 4:**
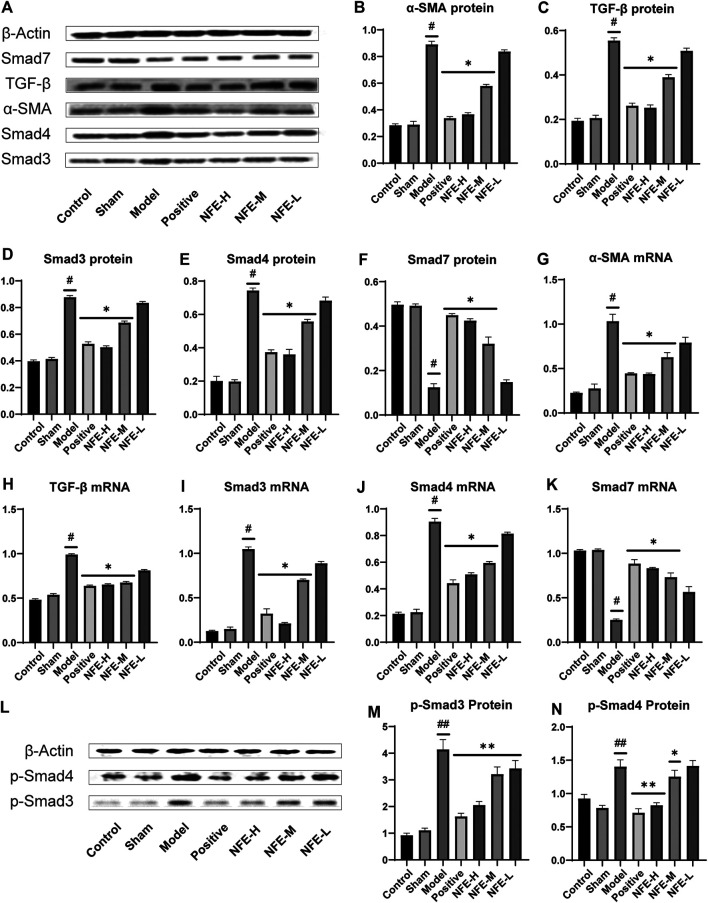
Effect of NFE by regulating the TGF-β/Smad pathway in rats. Protein and mRNA expression levels involved in the TGF-β pathway were determined by western blotting **(A,L)** and qRT-PCR; the protein levels of α-SMA **(B)**, TGF-β1 **(C)**, Smad3/4/7 **(D,E,F)** and *p*-Smad3/4 **(MN)**, and the mRNA expression of *a*-SMA **(G)**, TGF-β1 **(H)** and Smad3/4/7 **(I,J,K)** were regulated by NFE. #*p* < 0.05 compared with control group. ##*p* < 0.01 compared with control group. **p < 0.01 compared with model group. *p < 0.05 compared with model group.

To investigate whether the therapeutic efficacy of NFE in the BLM-induced model was due to the reduction in the TGF-β/Smad pathway signaling in rats, we investigated the effect of NFE on the expression of TGF-β1, Smad3/4/7, and *α*-SMA. Our results showed that treatment with NFE in BLM-induced rats significantly alleviated the progression of pulmonary fibrosis. Furthermore, based on our previous experience, the regulation of the TGF-β pathway may be considered a key influencing factor in the treatment process. As shown in Figure 4, the expression of TGF-β1 and *α*-SMA proteins was significantly upregulated in the lung tissues of BLM-induced pulmonary fibrosis rat model compared with that in normal control rats. NFE (NFE-H 400 and NFE-M 200 mg/kg) treatment significantly and dose-dependently downregulated TGF-β1 and *α*-SMA expression.

Smad3 and Smad4 proteins were overexpressed in BLM-induced pulmonary fibrosis rats compared with normal rats. NFE (400 mg/kg and 200 mg/kg) distinctly and dose-dependently downregulated Smad3 and Smad4 expression. Meanwhile, the expression levels of phosphorylation of Smad3 and Smad4 (*p*-Smad3/4) were significantly decreased in bleomycin-treated rats after treatment with NFE. In addition, BLM significantly reduced Smad7 protein expression compared with normal control rats, and NFE (400 and 200 mg/kg) significantly upregulated Smad7 expression.

The qRT-PCR results showed that 400 and 200 mg/kg of NFE treatment significantly increased or decreased mRNA expression of these proteins, respectively, in a dose-dependent manner compared with the BLM model group.

**FIGURE 5:**
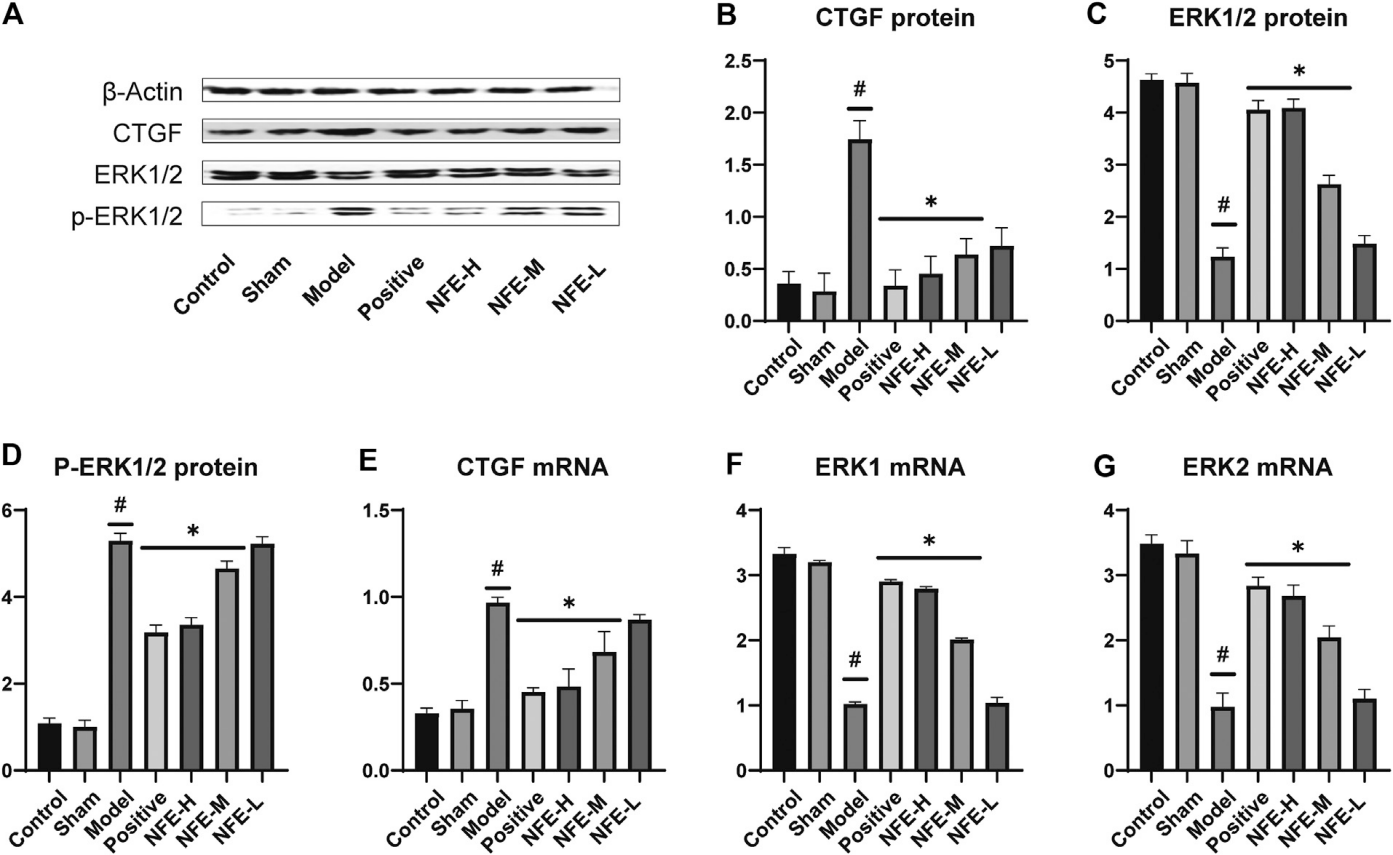
Effect of NFE by regulating the activation of CTGF and phosphorylation of ERK1/2 in rats. Protein expression levels of CTGF and downstream protein ERK1/2 were determined by western blotting **(A)** and qRT-PCR; the protein expression of CTGF **(B)**, ERK1/2 **(C)** and *p*-ERK1/2 **(D)**, and the mRNA expression of CTGF **(E)**, ERK1 **(F)** and ERK2 **(G)** were regulated by NFE. #*p* < 0.05 compared with control group. **p < 0.01 compared with model group. *p < 0.05 compared with model group.

The synthesis and deposition of collagen in the lung tissue can be caused by the activation of CTGF and phosphorylation of ERK. Western blotting analysis showed that CTGF protein levels were upregulated in BLM-induced pulmonary fibrosis rats, and NFE (400 and 200 mg/kg) treatment downregulated its expression levels (Figure 5). Similarly, the protein level of ERK1/2 was significantly downregulated, while that of *p*-ERK1/2 was significantly upregulated in BLM-induced pulmonary fibrosis rats. NFE (400 and 200 mg/kg) treatment significantly and dose-dependently normalized the phosphorylation of ERK1/2, upregulated ERK1/2 protein levels, and downregulated *p*-ERK1/2 protein levels.

At the level of phosphorylation of ERK protein, the results of mRNA analysis using qRT-PCR were similar to those of protein expression. The effects of NFE at 400 and 200 mg/kg resulted in a significant correction of the mRNA level of non-phosphorylated ERK1/2, with the downregulation of CTGF mRNA levels.

### NFE Alleviated LPS-Induced Pulmonary Fibrosis in Fibroblasts

**FIGURE 6:**
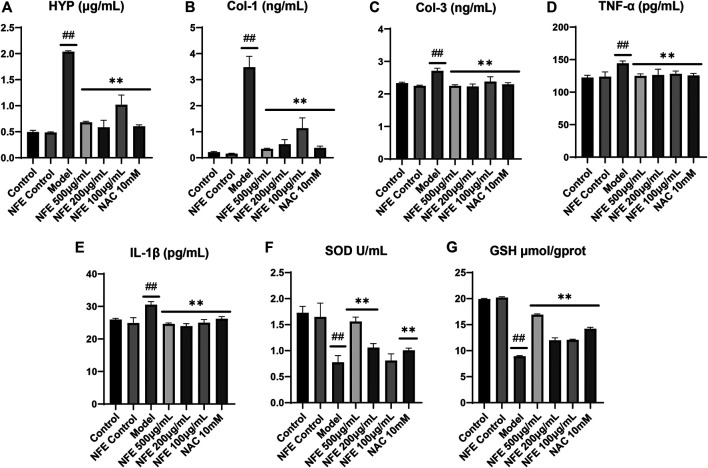
NFE alleviated LPS-induced pulmonary fibrosis in fibroblasts. The levels of HYP **(A)**, collagen-1 (Col-1) **(B)**, collagen-3 (Col-3) **(C)**, tumor necrosis factor-α (TNF-α) **(D)**, interleukin-1β (IL-1β) **(E)**, superoxide dismutase (SOD) **(F)** and GSH **(G)** were regulated by NFE. ##*p* < 0.01 compared with control group. **p < 0.01 compared with model group. *p < 0.05 compared with model group.

To directly prove the effects of NFE on pulmonary fibrosis, lung fibroblasts from mice (swiss-3T6) were evaluated. *In vitro* cell models were developed using LPS-induced 3T6 cells. We initially determined the anti-fibrotic, anti-oxidative, and anti-inflammatory effects of NFE. As expected, compared with the control group, significantly higher levels of HYP, Col-1, and Col-3 were observed in LPS-induced cells, whereas NFE administration attenuated LPS-induced HYP content by 1 to 2-fold (Figure 6A–C). Inflammation played a critical role in the progression of pulmonary fibrosis. Fibroblasts stimulated by LPS excessively secreted cytokines, such as TNF-α and IL-1β (Figure 6D,E), however, cytokine secretion was decreased after NFE treatment. Similarly, the inhibitory effect of NFE was confirmed by the upregulation of SOD and GSH levels by LPS (Figure 6F,G), a reflection of its ability to resist oxidative damage.

### Effect of NFE on Cell Migration of Fibroblasts

**FIGURE 7:**
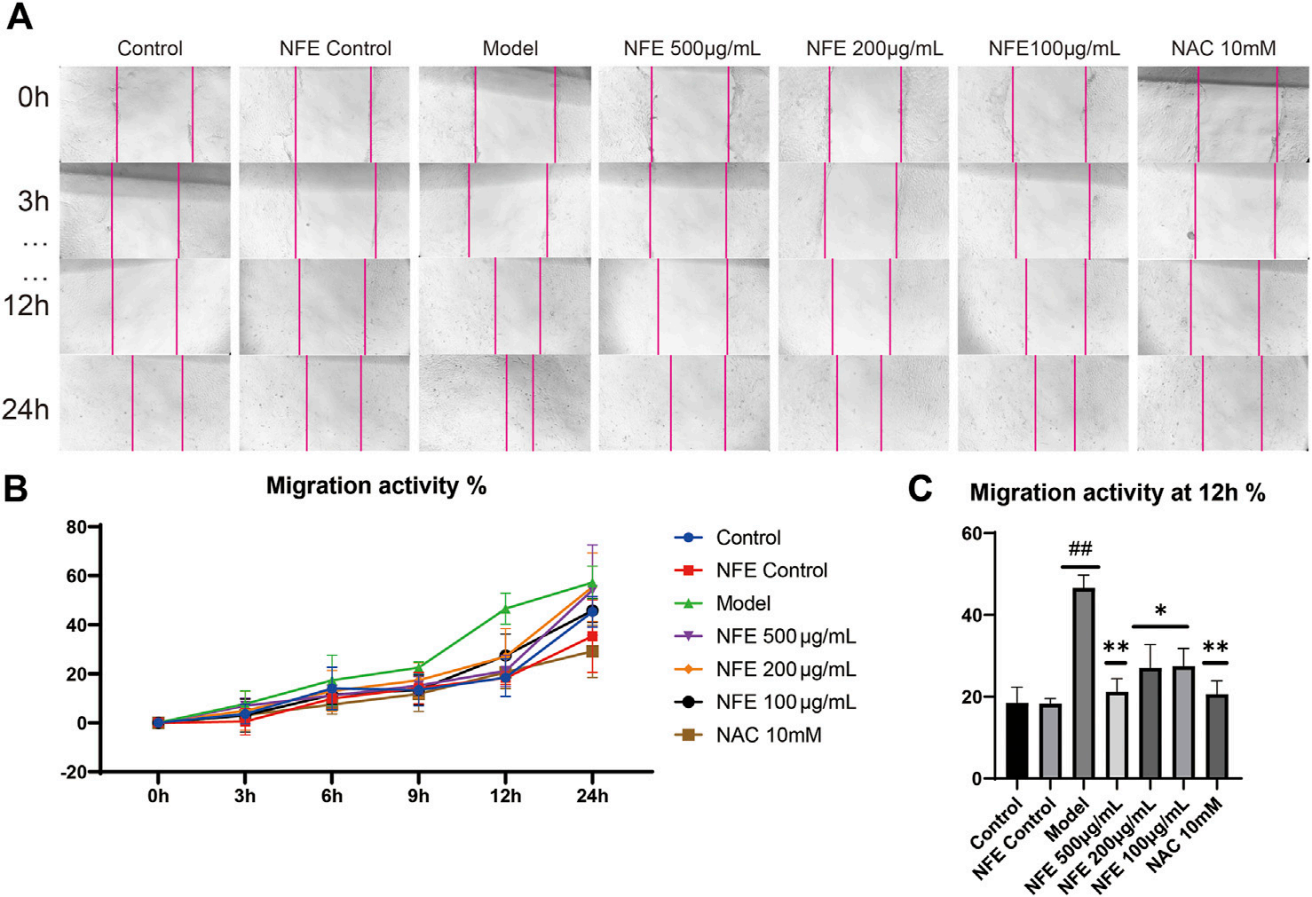
NFE inhibited the migration of fibroblasts. The effect of NFE on cell migration was assessed using scratch wound assay **(A)**, and the wound closure was photographed at post-scratching 0, 3, 6, 9, 12, and 24 h **(B)** shows typical cases at 0, 3, 12, and 24 h, and **(C)** shows the migration activity at 12 h ##*p* < 0.01 compared with control group. **p < 0.01 compared with model group. *p < 0.05 compared with model group.

Furthermore, we determined whether NFE was capable of inhibiting fibroblast migration *in vitro*. Migration activity was assessed using a scratch wound closure assay, in which 3T6 cells were cultured with LPS, NFE, or NAC for 24 h. Images of the migrated cells were captured using a light microscope, and wound closure was photographed at 3, 6, 9, 12, and 24 h post-scratching (representative images are shown in Figure 7A,B). Wound healing effects of LPS were determined by calculating the wound closure (%) by measuring the wound area. It was found that LPS increased the rate of wound closure after 12 h, reinforcing the proliferative and invasive capacity of cells *in vitro*. Under similar conditions, NFE treatment considerably inhibited cellular migration (Figure 7C). This result suggested that the anti-fibrotic activity of NFE contributed to the inhibition of fibroblast migration and effectively prevented fibrosis formation.

### Effect of NFE by Regulating the TGF-β/Smad Pathway in Fibroblasts

**FIGURE 8:**
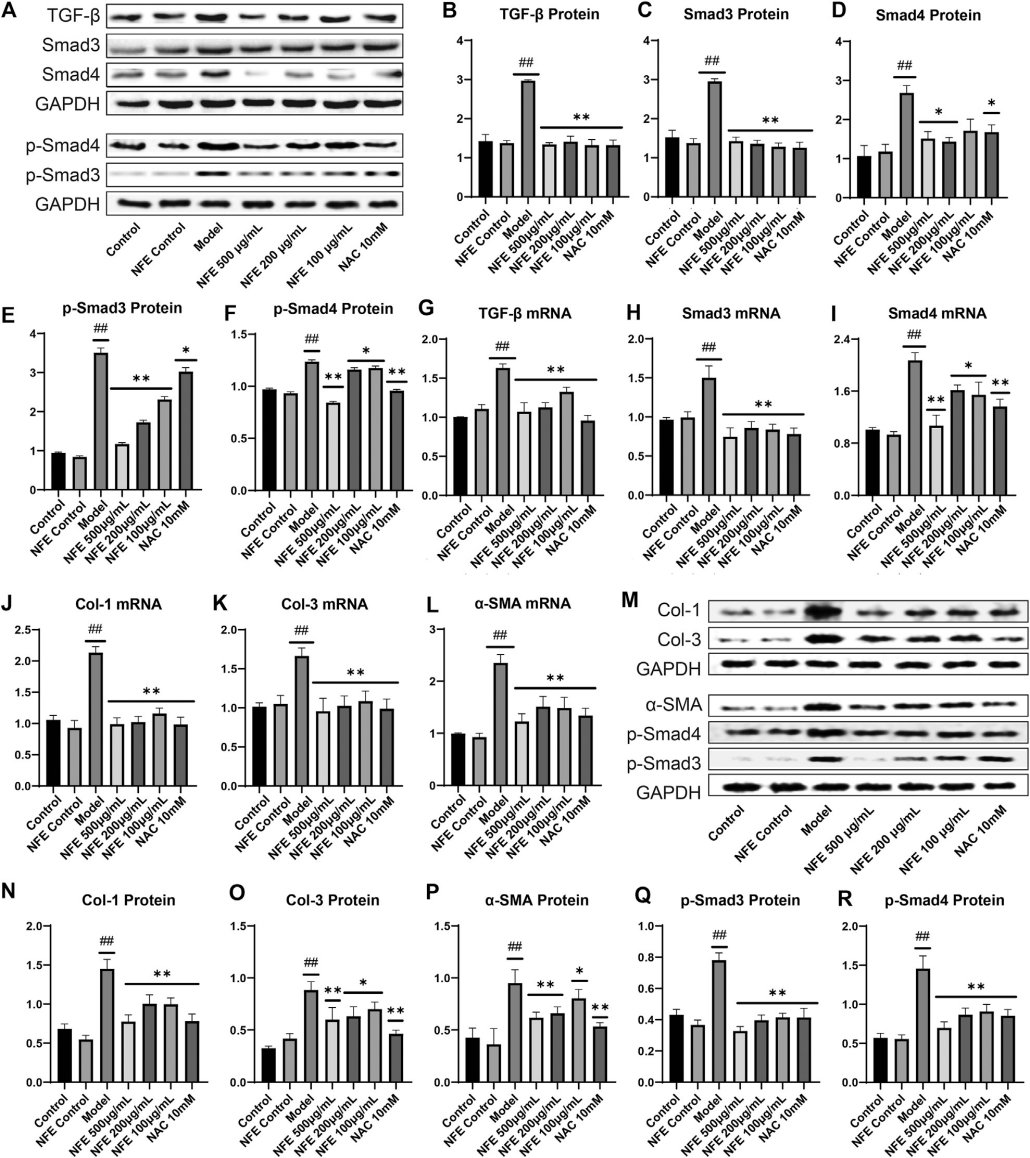
Effect of NFE by regulating the TGF-β/Smad pathway in LPS-induced and TGF- β1-induced fibroblasts. As for LPS-induced fibroblasts, protein and mRNA expression levels were determined by western blotting **(A)** and qRT-PCR. The protein levels of TGF-β1 **(B)**, Smad3/4 **(C,D)** and *p*-Smad3/4 **(E,F)**, and the mRNA expression of TGF-β1 **(G)**, Smad3 **(H)** and Smad4 **(I)** were regulated by NFE; Likewise, the mRNA expression of Col-1 **(J)**, Col-3 **(K)** and α-SMA **(L)** were also determined. As for TGF-β1-induced fibroblasts, protein expression levels were determined by western blotting **(M)**, including Col-1 **(N)**, Col-3 **(O)**, *a*-SMA **(P)**, *p*-Smad3/4 **(Q,R)**. ##*p* < 0.01 compared with control group. **p < 0.01 compared with model group. *p < 0.05 compared with model group.

**FIGURE 9:**
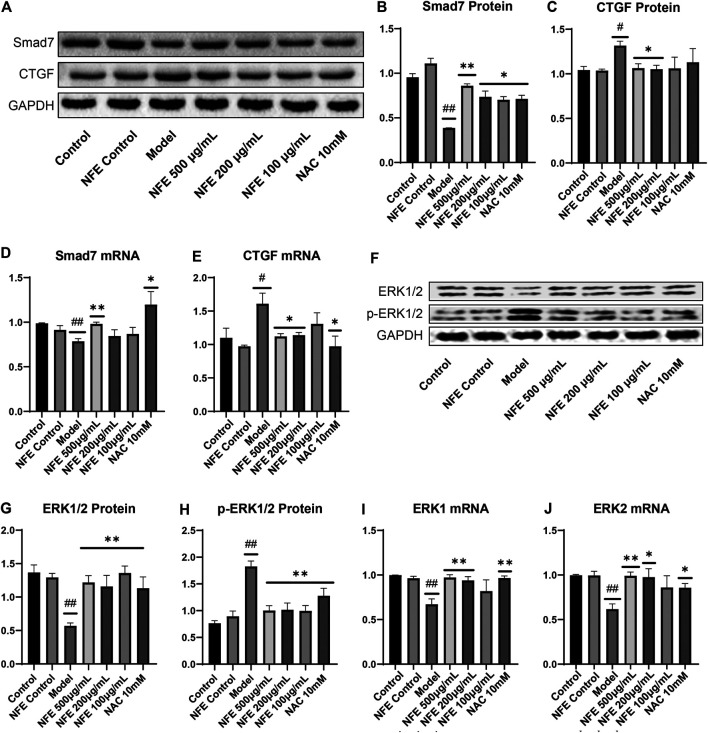
Effect of NFE by regulating the activation of CTGF and phosphorylation of ERK1/2 in fibroblasts. Protein and mRNA expression levels were determined by western blotting **(A,F)** and qRT-PCR. The protein levels of Smad7 **(B)**, CTGF **(C)**, ERK1/2 **(G)** and *p*-ERK1/2 **(H)**, and the mRNA expression of Smad7 **(D)**, CTGF **(E)**, ERK1 **(I)** and ERK2 **(J)** were regulated by NFE; ##*p* < 0.01 compared with control group. **p < 0.01 compared with model group. *p < 0.05 compared with model group.

To further substantiate the role of NFE in the TGF-β pathway, we analyzed the expression of TGF-β1 and other related genes in fibroblasts stimulated by LPS with or without NFE. Western blotting and qRT-PCR analysis indicated high levels of TGF-β1 protein and mRNA expression in the model group, while NFE and NAC treatment decreased the production and secretion of TGF-β1 (Figure 8A,B,G). TGF-β mediates fibrosis effects on most target cells via activation of the canonical SMAD signaling pathway, thus, we measured the levels of Smad3/4 and Smad7 upon LPS, NFE, or NAC stimulation. As expected, an increase in Smad3/4 protein and mRNA expression was observed in the model group (Figure 8C,D,H,I), while the expression of Smad7 decreased (Figure 9A,B,D). Additionally, the expression levels of *p*-Smad3 and *p*-Smad4 were significantly decreased in LPS-induced fibroblasts after treatment with NFE (Figure 8E,F). These results indicated that NFE inhibited the activation of TGF-β/Smad signaling pathway, and NFE antagonized the effects of LPS on fibroblasts.

Because our data demonstrate that inhibition of TGF-β1 activity was sufficient to reduce the expression of fibrosis-related genes in rats, such as *a*-SMA and collagen, we questioned whether it was also necessary to evaluate their mRNA levels in fibroblasts. qRT-PCR analysis of fibroblasts showed an increase in the mRNA expression of Col-1, Col-3, and *α*-SMA, whereas their expression was reduced after NFE administration (Figure 8J,K,L). To futher understand the anti-fibrosis effect of NFE *in vitro*, we tested the protein expression of fibroblasts induced by TGF-β1, as shown in Figure 8 M to R. The protein expression levels of Col-1, Col-3 and *α*-SMA were significantly increased in the model group. After the intervention of NFE, those protein expressions levels were significantly reduced. Consistent with the previous study, the over-expression of phospho-SMAD (*p*-Smad3/4) protein induced by TGF-β1 was also significantly decreased by NFE. The above results confirmed that NFE alleviated fibrosis induced by TGF-β1, and suppressed Smad3/4 phosphorylation *in vitro*.

TGF-β is known to positively regulate CTGF expression through Smad activation in fibroblasts and induces fibrosis *in vitro* and *in vivo*. Treatment with NFE reduced LPS-induced CTGF protein and mRNA expression (Figure 9C,E). Consistently, activation (phosphorylation) of ERK has been reported to enhance TGF-β/Smad mediated responses. We further assessed the protein and mRNA expression of ERK1 and ERK2, and similar results were obtained (Figure 9F–J). Collectively, these data suggest that NFE inhibits TGF-β/Smad signaling and has a potent antifibrotic effect.

## Discussion

Pulmonary fibrosis is a fatal lung disease characterized by the accumulation of ECM as a result of lung remodeling. The pathogenesis of pulmonary fibrosis may be instigated by injuries of lung epithelial cells via fibrogenic stimuli, which is important for lung inflammation and controlled by several types of cells and cytokines.

LPS, an endotoxin from Gram-negative bacteria, is a potent inducer of fibrosis progression and inflammatory cytokines. BLM is a glycopeptide antibiotic conventionally used in anticancer therapy, and one of its side effects is isolated pulmonary inflammation and fibrosis, as supported by lung histopathological indices and an increase in various inflammatory and oxidative stress indices. In the present study, we used the classic method of intratracheal instillation of BLM to develop a pulmonary fibrosis model of rats, while the swiss-3T6 fibroblast cells were induced by LPS for the migration, inflammation, and fibrosis processes with the intervention of NFE.

Previous experimental studies have shown that the TGF-β/Smad signaling pathway is activated after BLM, LPS and TGF-β1 stimulation. The protective effect of NFE on BLM-induced pulmonary fibrosis rats and LPS/TGF-β1-induced fibroblasts by observing pathological changes, migration activity, and related protein and mRNA expression was investigated. NFE significantly reduced the severity of inflammation and delayed the development of pulmonary fibrosis, in which the administration of 400 mg/kg of NFE to rats resulted in better results.

TGF-β plays a key role in the progression of pulmonary fibrosis by promoting proliferation and differentiation of fibroblasts, enhancing collagen synthesis, and altering some components of the ECM. Smad proteins were the main signal transducers of the TGF-β signaling pathway, in which Smad3 binds to Smad4, forms a complex and enters the nucleus to regulate the targeted gene transcription, which thereby interferes with the formation of lung fibrosis (Yamazaki et al., 2017). On the contrary, Smad7 inhibits pulmonary fibrosis by competitively binding to TGF-β and its receptors complex with Smad3 (Zaringhalam et al., 2014; Yan et al., 2016).

**FIGURE 10:**
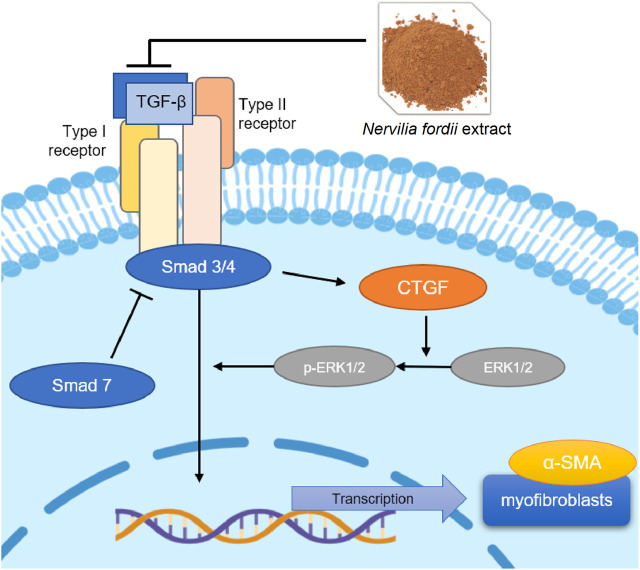
TGF-β/Smad mediated signaling pathways affected the NFE treatment of BLM-induced or LPS-induced pulmonary fibrosis.

During this process, CTGF is activated because of a significant increase in TGF-β expression, regulation of related proteins, and massive proliferation of lung fibroblasts (Nakai et al., 2019). Collagen fibers are deposited in large amounts in the lung tissue, resulting in the substantialization of lung tissue. At the same time, overexpression of CTGF rapidly causes phosphorylation of the downstream protein ERK1/2 to generate *p*-ERK1/2 (Song et al., 2019), which accelerates the deterioration of pulmonary fibrosis symptoms. *α*-SMA is an interstitial marker protein secreted by myofibroblasts and is the main indicator of ECM content. These mechanisms are clearly shown in Figure 10.

After 14 days, it was discovered that the protein and mRNA expression of TGF-β1, *α*-SMA, Smad3/4, and *p*-Smad3/4 were significantly downregulated after the treatment with NFE, while the protein and mRNA expressions of Smad7 were significantly upregulated. Considering that NFE is a traditional Chinese medicine extract rather than a powerful active compound, we chose to initiate the drug intervention only 1 day after bleomycin instillation, in order to initially investigate the potential of NFE to intervene in acute lung injury to delay pulmonary fibrosis. Our results showed that NFE could reduce acute lung injury induced by BLM, these findings may show the potential effect of NFE in the treatment of fibrosis. Due to the alleviation of inflammation and lung damage, the occurrence and development of fibrosis were prevented in the first place. The results of the *in vitro* experiments were consistent with those of *in vivo* experiments. The cell model of LPS-induced fibroblasts simulates the therapeutic effect of NFE on the fibroblast-to-myofibroblast differentiation in an inflammatory environment *in vitro*. And the further results showed the anti-fibrosis effects of NFE on TGF-β1-induced fibroblasts. The present study suggests that NFE could ameliorate the progression of IPF by inhibiting the TGF-β/Smad pathway. Furthermore, we would further determine whether NFE can be used in therapeutically treat established fibrosis models in future experiments, and continue to explore the direct active compounds of NFE.

In summary, the present study demonstrates the effect of *N. fordii* extract (NFE) on pulmonary fibrosis, involving TGF-β/Smad mediated signaling pathways, activation of CTGF, and phosphorylation of ERK1/2. This result indicates that the flavonoids of traditional Chinese herbal medicines have good prospects for the treatment of pulmonary fibrosis.

## Data Availability

The original contributions presented in the study are included in the article/Supplementary Material, further inquiries can be directed to the corresponding authors.
