# Supplementary material for: Effects of Nervilia fordii Extract on Pulmonary Fibrosis Through TGF-β/Smad Signaling Pathway
Source: Front Pharmacol. 2021 Apr 19;12:659627. doi: 10.3389/fphar.2021.659627 (PMC8090936; doi:10.3389/fphar.2021.659627)
Supplement: Supplementary file 1 [file datasheet1.docx]

Supplementary Material

**Supplementary materials (Part A)**

**The qualitative and quantitative analysis of NFE**

**1. Materials and method**

**1.1. Chemicals and reagents**

Rhamnocitrin 3,4'-O-glucoside (**1**, PubChem CID 5492406), nervilifordin D (**2**, PubChem CID 44179499), nervilifordin B (**3,** PubChem 44179423), rhamnazin 3-O-glucopyraconoside **(4,** PubChem CID 14704550), and rhamnocitrin (**5**, PubChem CID 5320946) were isolated from *Nervilia fordii,* and their structures were identified using UV, IR, MS, and NMR (^1^H-NMR and ^13^C-NMR) (Zhang et al., 2012a;Zhang et al., 2012b). The purity of each of the above five standard substances was more than 99%. This was done by normalizing the peak area determined using HPLC. Deionized water was purified using a Millipore water purification system (Millipore, Billerica, MA, USA). The acetonitrile and methanol used in the study were all pure grade UPLC-MS (Fisher Chemical Company, Geel, Belgium). Formic acid was purchased from Sigma-Aldrich. Other analytical grade reagents were purchased from the Guangzhou Chemical Reagent Factory (Guangzhou, China).

**1.2. Qualitative identification of NFE by UPLC-Q-TOF-MS**

UPLC-Q-TOF-MS analysis was performed using a Shimadzu UPLC LC-30AD system (Shimadzu, Kyoto, Japan) coupled to a tandem MS AB SCIEX TripleTOF 5600+ (AB SCIEX, Foster City, CA, USA). Chromatographic separations of processed samples were done on an Agilent C18 column (3.0 × 50 mm, 2.7 μm, Agilent Technologies Inc., USA). Column temperature was maintained at 40 °C. The autosampler was maintained at 4 °C. The mobile phase consisted of (A) acetonitrile and (B) water containing 0.1% formic acid using a gradient elution as follows: 15%–55% A at 0–7 min, 55%–85% A at 7–9 min. The flow rate was 0.4 mL/min and the injection volume was 2 μL.

For mass spectrometric detection, an electrospray ionization source (ESI) interface was set in both positive and negative modes (to monitor as many ions as possible). The ion spray voltage was set to 4,500 V; the turbo spray temperature was 550°C; nebulizer gas (Gas 1), 55 psi; heater gas (Gas 2), 55 psi; and declustering potential (DP) 100V, the spectra covered the range from m/z 100–1000 Da.

All data were processed using the Analyst Software^TM^ 2.2 (AB SCIEX, Foster City, CA, USA). Post-acquisition analyses were performed using AB SCIEX PeakView^TM^ (v2.1) software and MasterView^TM^ (v1.0) software.

**1.3. Quantitative analysis of 5 main components in NFE by UPLC-MS**

UPLC-MS analysis was performed using an Agilent 6460 triple quadrupole LC-MS system equipped with ESI. Chromatographic separation was done on an Agilent SB-C18 column (3.0 × 50 mm, 2.7 μm). The column was maintained at 25°C, and the injection volume of the reference solution or sample solution was 5 μL. The mobile phase consisted of an aqueous solution containing acetonitrile (A) and 0.1% acetic acid (B). The column was eluted at a flow rate of 0.4 mL/min using the following gradient: 0–1 min, 20%-30% A, 1–3 min, 30%-60% A, 3–4 min, 60%-70% A. The mobile phase was diverted before MS analysis.

The parameters for the ionization source conditions were as follows: spray voltage, 3500 V; capillary temperature, 300°C; and vaporizer temperature, 300°C. Nitrogen was used as the Aux gas, and the sheath gas pressure was set at 45 psi. The scanning range of the mass spectrum was set at m/z 100–1000. Quantification was performed using multiple reaction monitoring (MRM) modes. Data acquisition and processing were performed using Xcalibar 1.4 Workstation.

**2. Results**

## 2.1. Chemical composition of NFE

Identification of the chemical constituents in NFE was performed using UHPLC-Q-TOF-MS/MS. In the full scan mass spectra, most of the identified compounds exhibited [M - H]- in the negative mode. The total ion current (TIC) chromatogram is shown in Figure S1. Multiple approaches, including database search, reference standard comparison, MS/MS spectrum analysis, and reasonable fragmentation pathways were employed for structural characterization of the constituents of NFE. A total of 20 compounds were identified, and all information concerning the identified compounds was summarized in Table S1 (A) and (B).

## 2.2 Contents of five main components in NFE

### 2.2.1. Methodology

### 2.2.1.1. Calibration curves, limits of detection, and limits of quantification

The mixed standard solutions of the five components were prepared in methanol as follows: rhamnocitrin 3,4'-O-glucoside 40 μm/mL, nervilifordin D 10 μm/mL, nervilifordin B 2 μm/mL, rhamnazin 3-O-glucopyranoside (0.1 μm/mL), and rhamnocitrin (0.2 μm/mL), and diluted to gradient concentrations to establish the calibration curves. At least six different concentrations were analyzed in triplicate, and the calibration curves were constructed by plotting the peak areas versus the concentration of each analyte. As shown in Table S2, all analytes showed good linearity (R2 ≥ 0.9990) over a relatively wide concentration range. The analysis of limit of detection (LOD), linearity, and limit of quantification (LOQ) also showed good quantification.

### 2.2.1.2. Precision, repeatability, and stability

Intra- and inter-day variations were further investigated to determine the precision of the developed method. To further evaluate the repeatability of the developed assays, samples were analyzed in six replicates, as described above. The stability of the samples was tested at room temperature and analyzed at different time points within one day. The contents of the five analytes were calculated from the corresponding calibration curves. Table S3 indicates that the RSD values for measurement precision, repeatability, and stability of the five analytes were all less than 5%, which demonstrates good precision, repeatability, and stability of the developed method.

### 2.2.1.3. Accuracy

Samples were analyzed in six replicates, as described above. The accuracy of the analytical method was evaluated by measuring the percentage recovery of the five compounds. The results of the recovery test are shown in Table S4, which ranged from 95% to 105%.

### 2.2.2. Quantification of the five components in NFE

The optimized and validated method was used for quantification of the five components in NFE. The MRM chromatograms of NFE are shown in Figure S2, and the MRM ion pair transitions and collision energy levels (CE) of each component are listed in Table S5. The contents of rhamnocitrin 3,4’-O-glucoside, nervilifordin D, nervilifordin B, rhamnazin 3-O-glucopyranoside, and rhamnocitrin in NFE were 42.59 ± 1.83 mg/g, 13.12 ± 0.40 mg/g, 0.79 ± 0.05 mg/g, 0.25 ± 0.06 mg/g, and 0.38 ± 0.02 mg/g (n=3), respectively.

**3. Conclusion**

Twenty compounds of NFE were qualitatively identified in this study, and five components were quantitatively determined, which will help us to further study the effective chemicals of this herb.

**Supplementary materials (Part B)**

**The MS/MS chromatograms by LC-Q-TOF-MS/MS of 20 compounds**

**No. name formula mass**

1 Nervilifordin E C34H42O22 802.2168


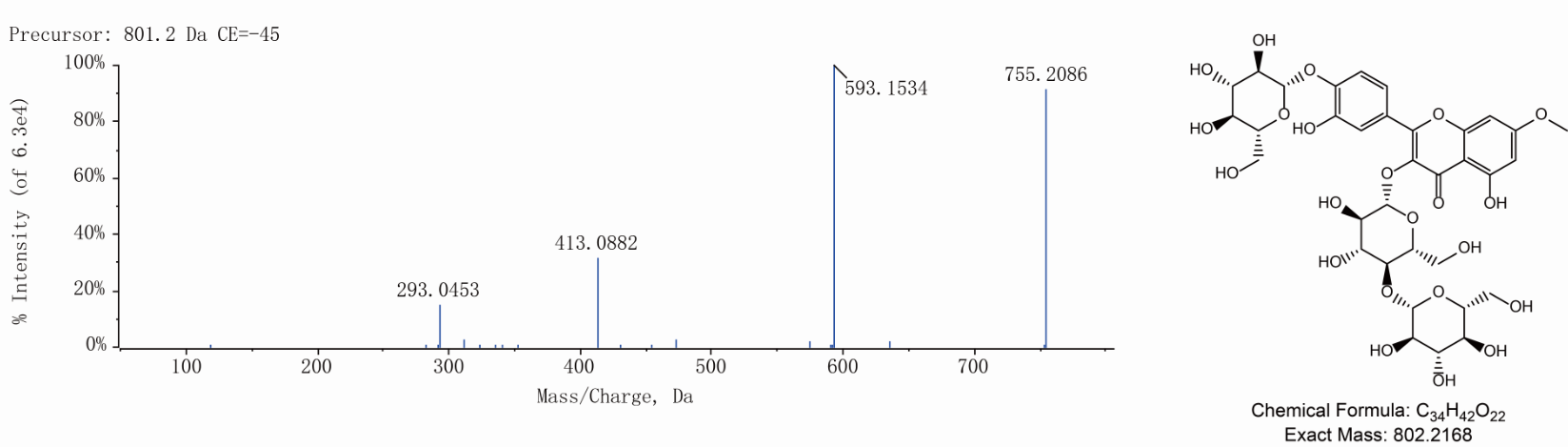


2 Nervilifordin A C27H30O15 594.1585


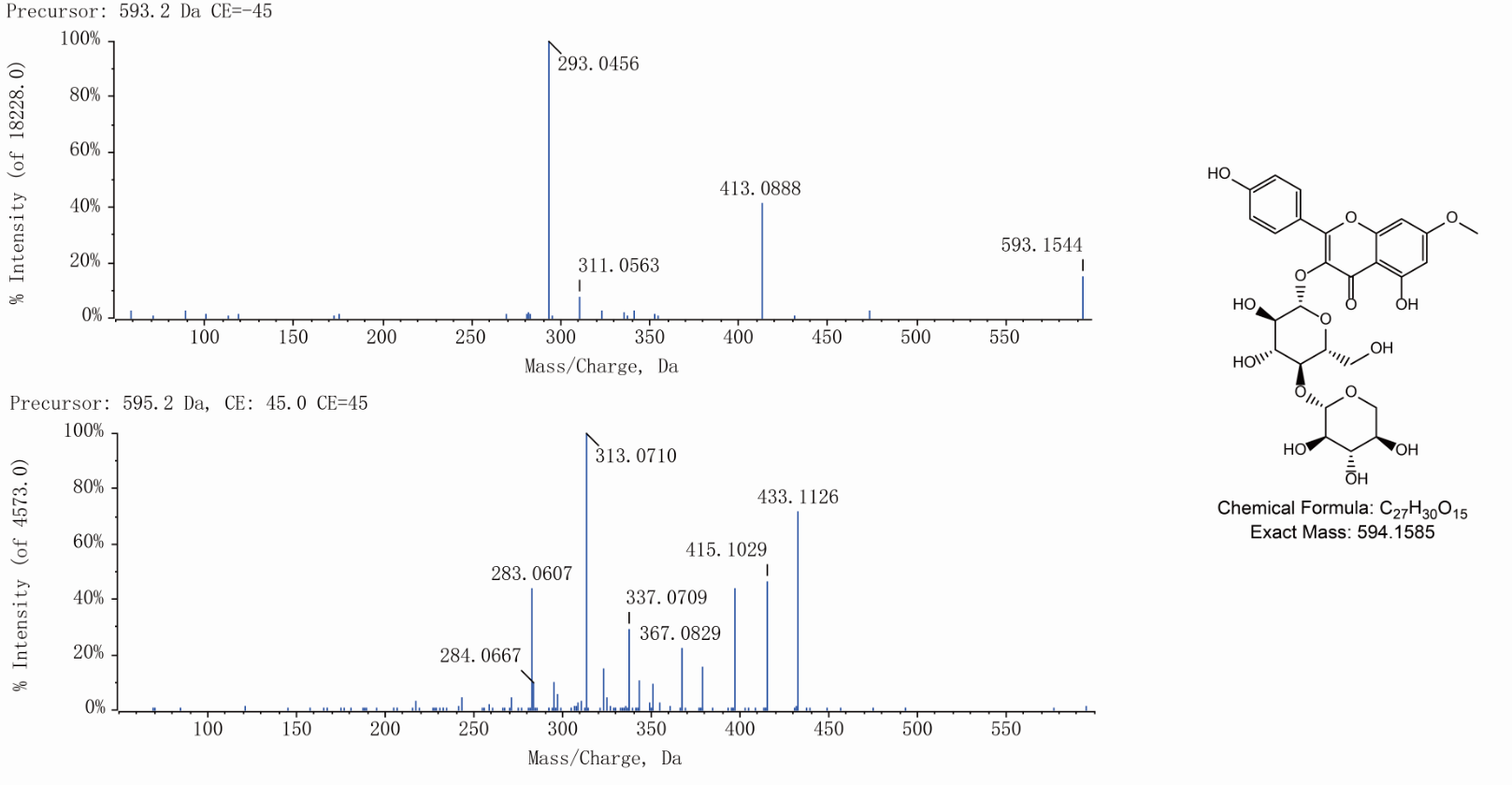


3 Apigenin 8-C-glucoside C21H20O10 432.1057


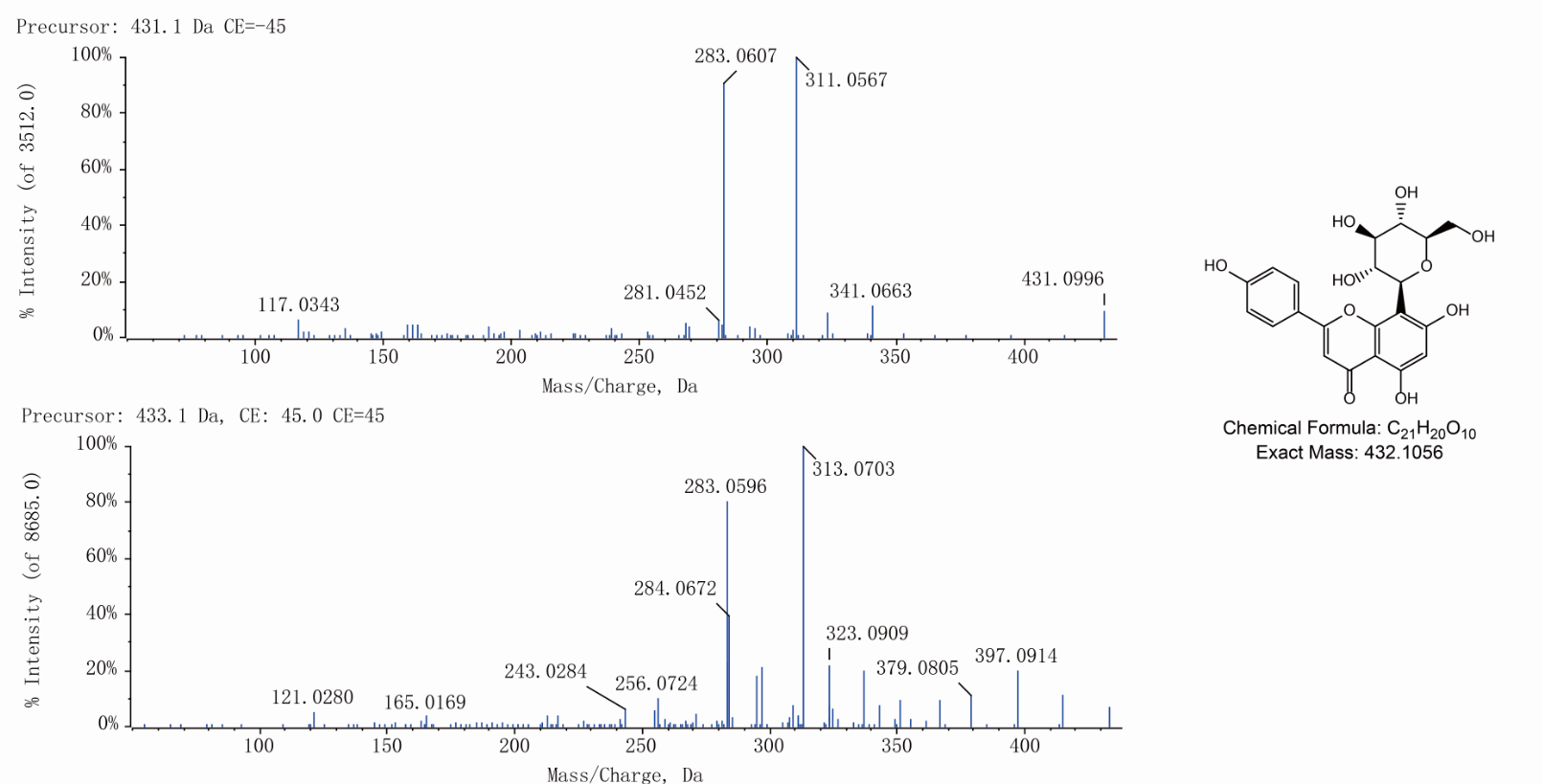


4 Nervilifordin D C34H42O21 786.2219


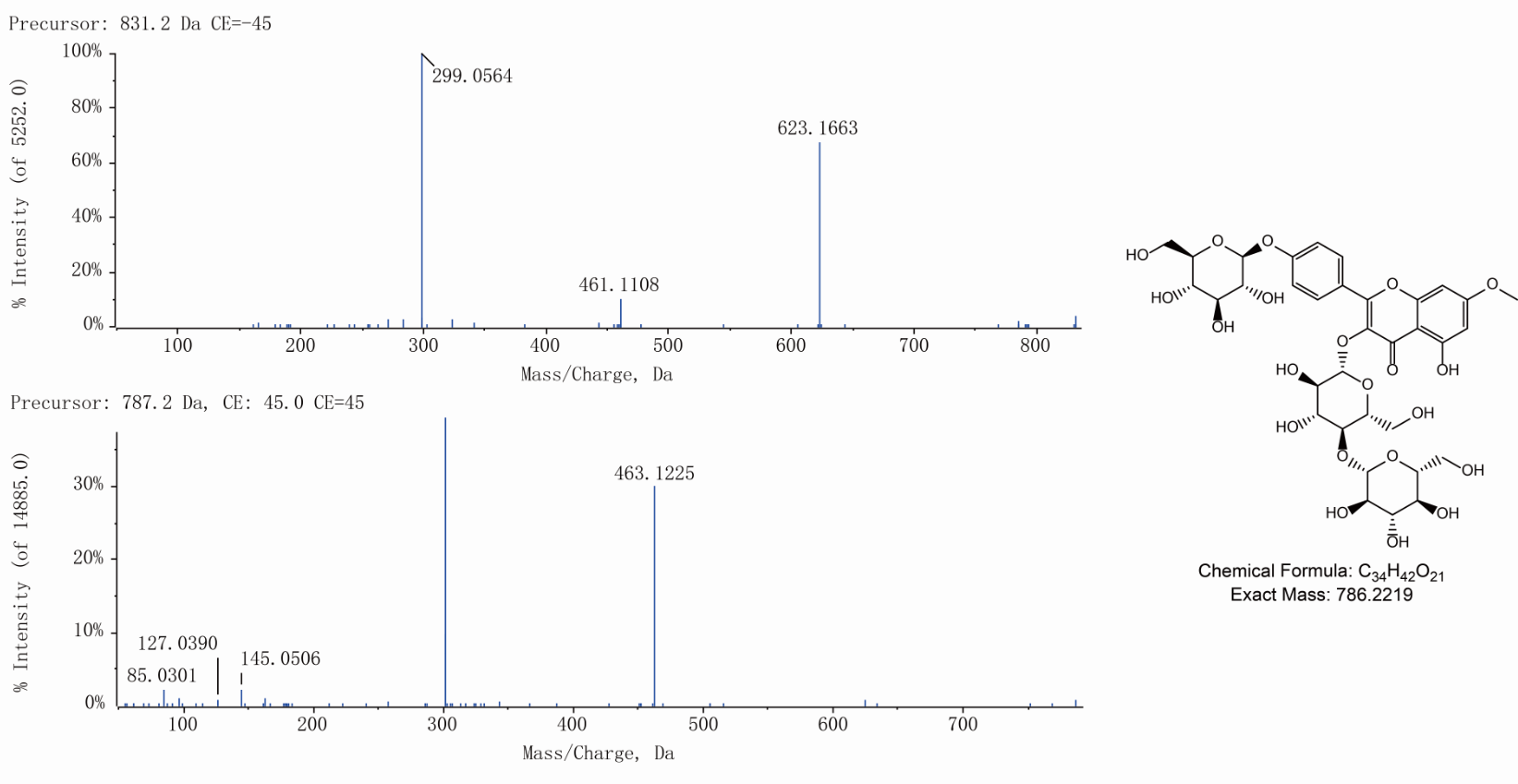


5 Nervilifordin C C28H32O17 640.1640


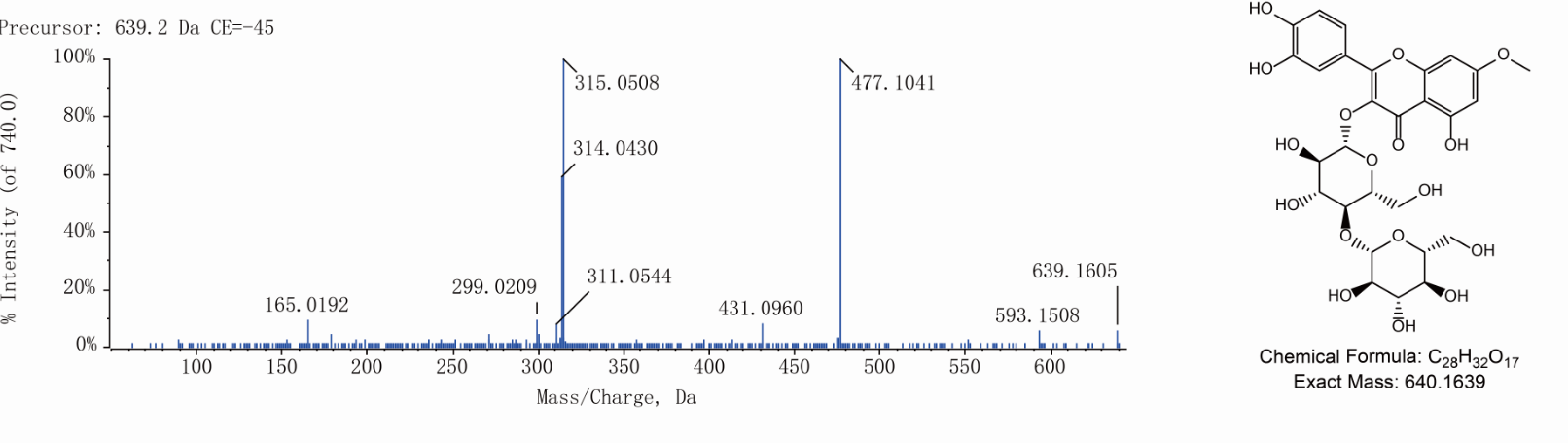


6 Rhamnocitrin 3,4'-O-glucoside C28H32O16 624.1690


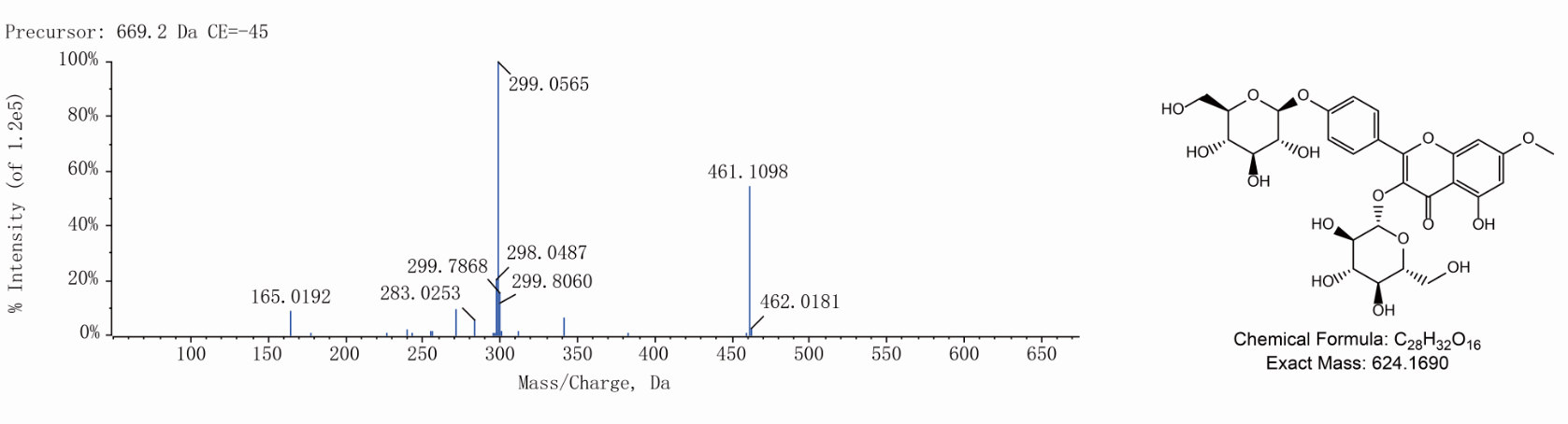


7 Rhamnazin 3,4'-O-glucoside C29H34O17 654.1796


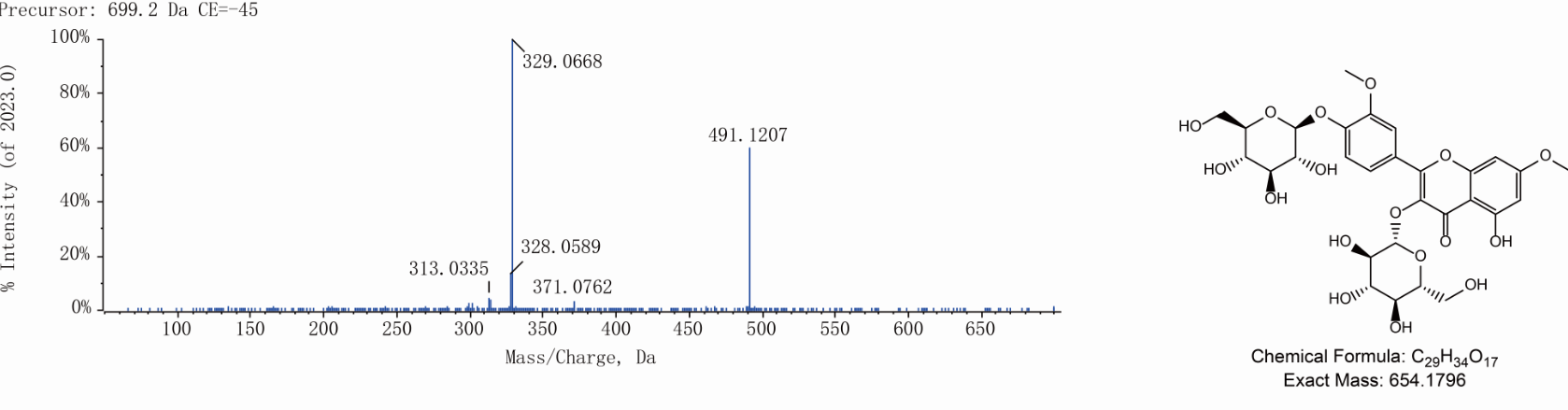


8 Nervilifordin B C28H32O16 624.1690


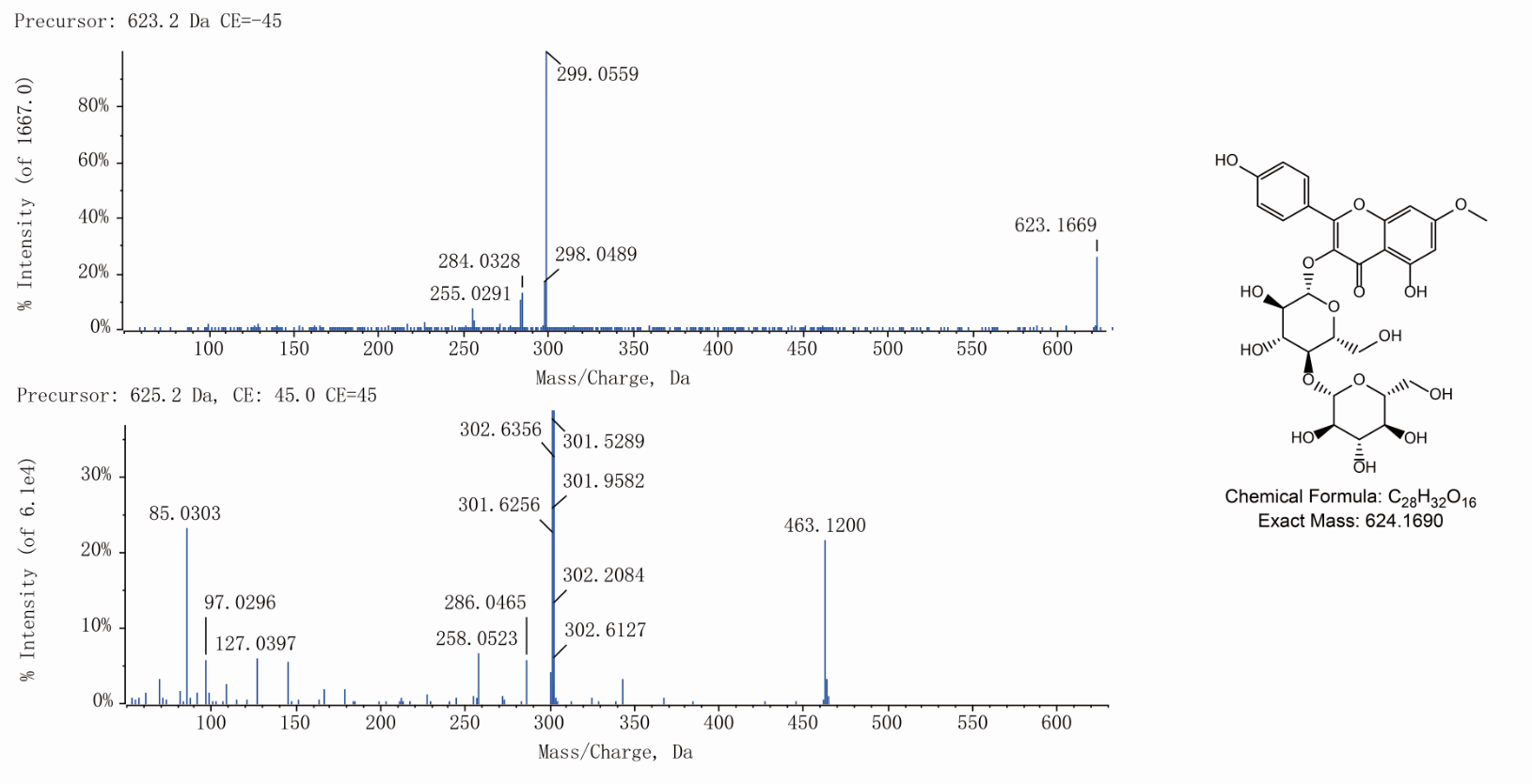


9 Rhamnocitrin 3-O-glucoside C22H22O11 462.1162


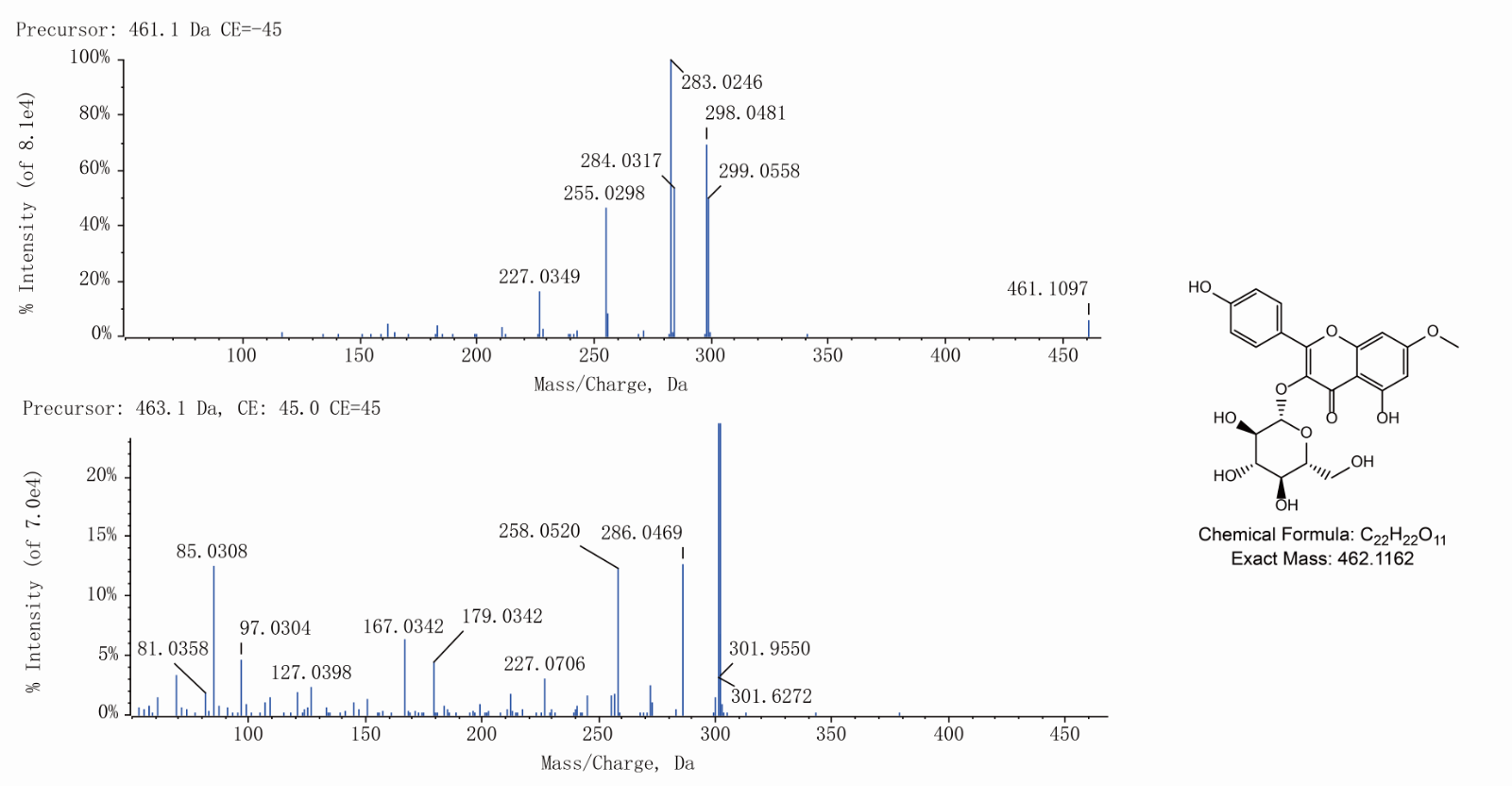


10 Rhamnazin 3-O-glucopyranoside C23H24O12 492.1268


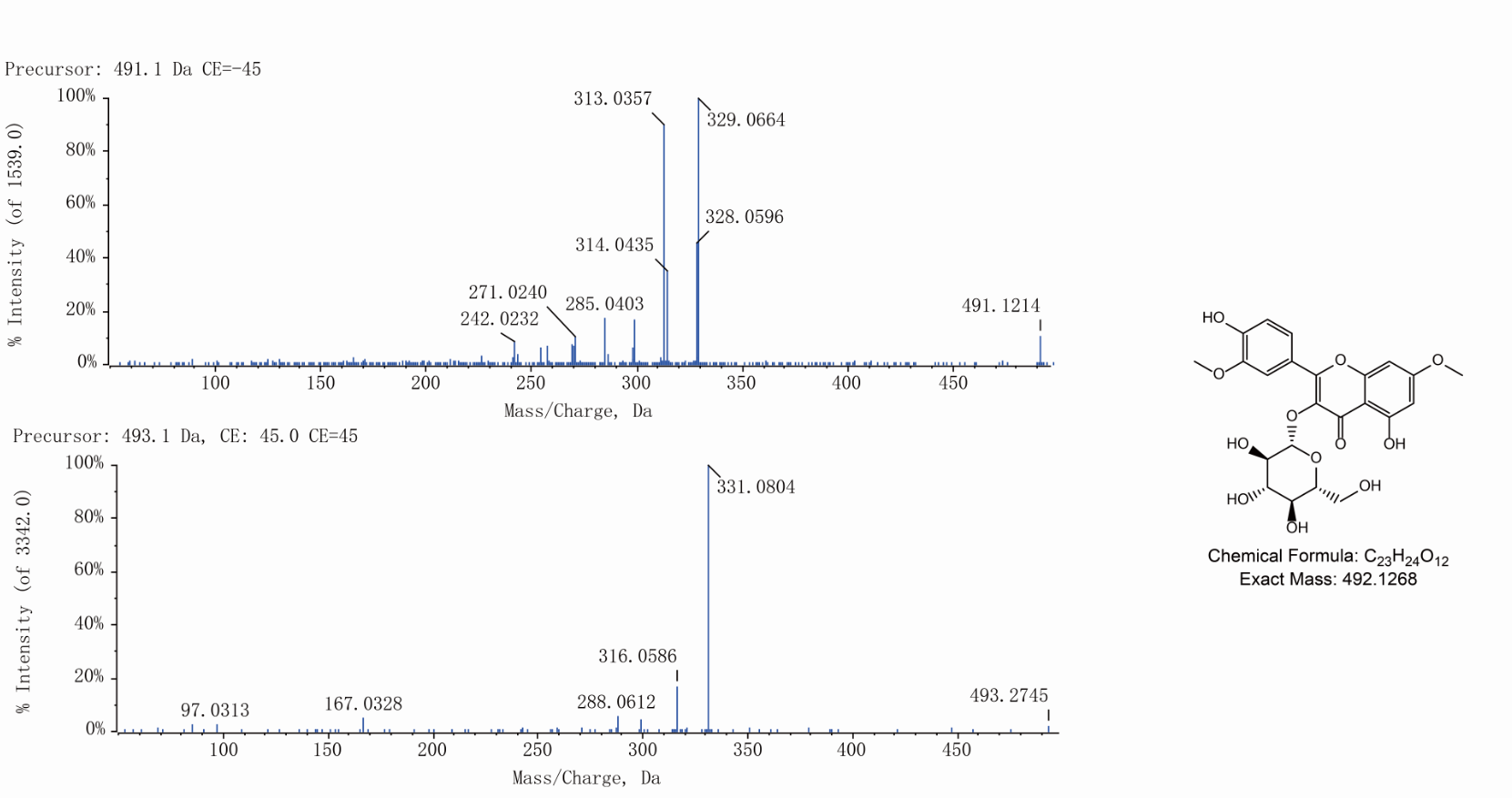


11 Rhamnazin C17H14O7 330.0740


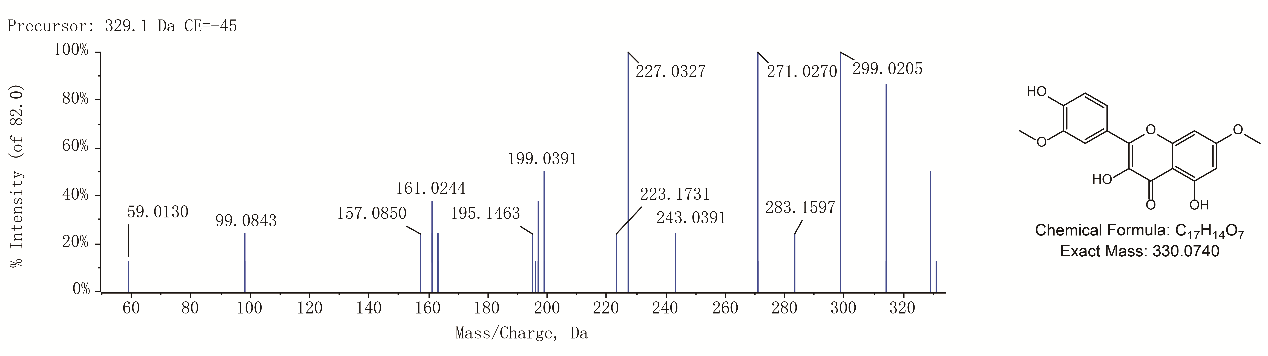


12 Rhamnetin C16H12O7 316.0583


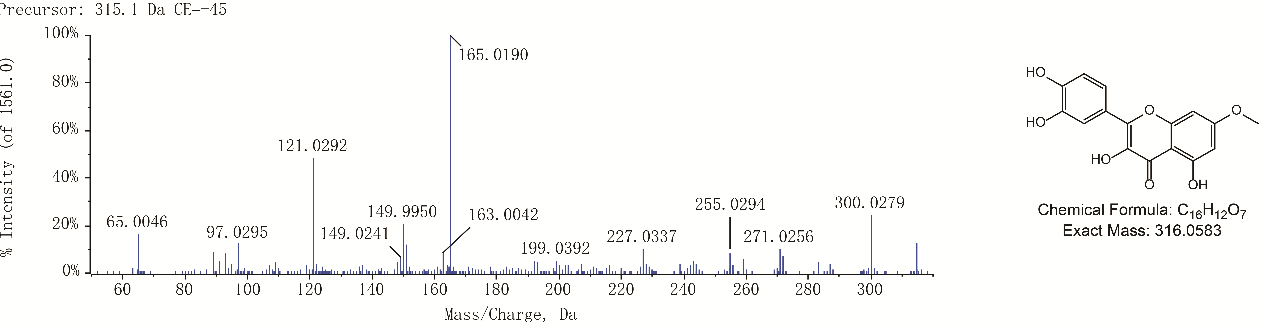


13 Rhamnocitrin C16H12O6 300.0634


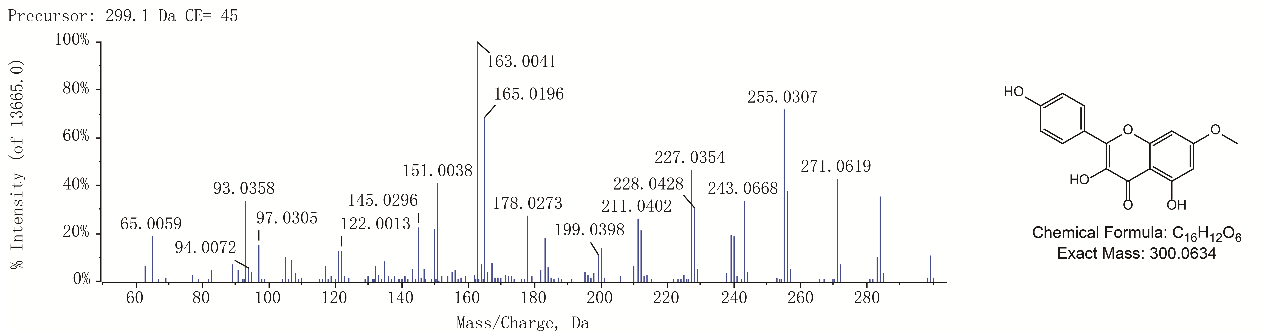


14 3-O-Caffeoylquinic acid C16H18O9 354.09508


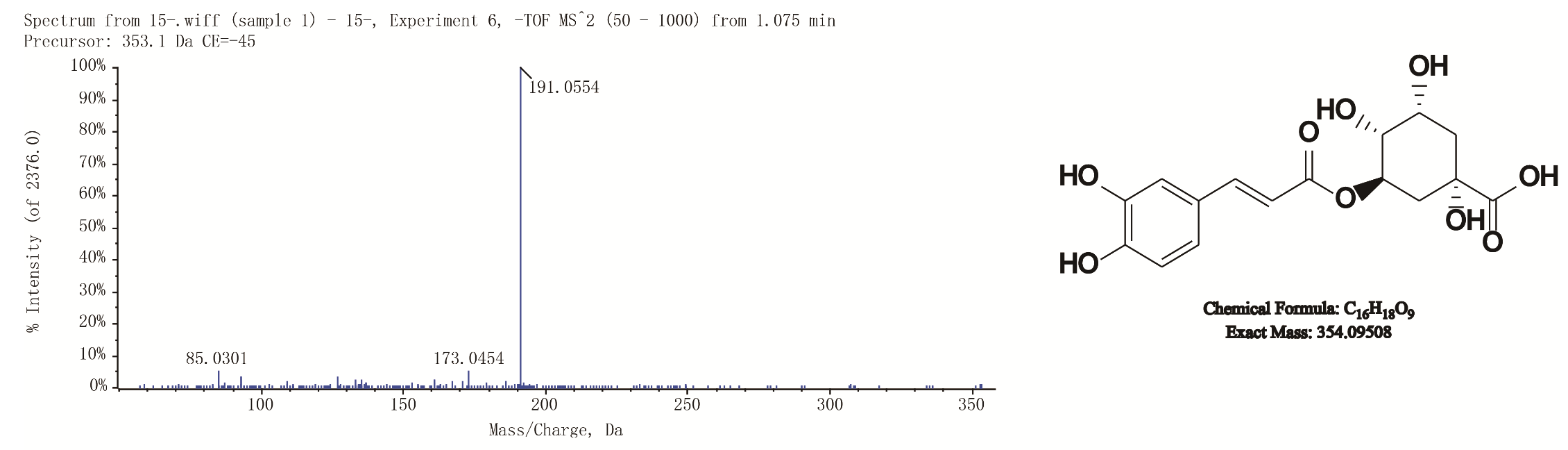


15 Esculetin C9H6O4 178.02661


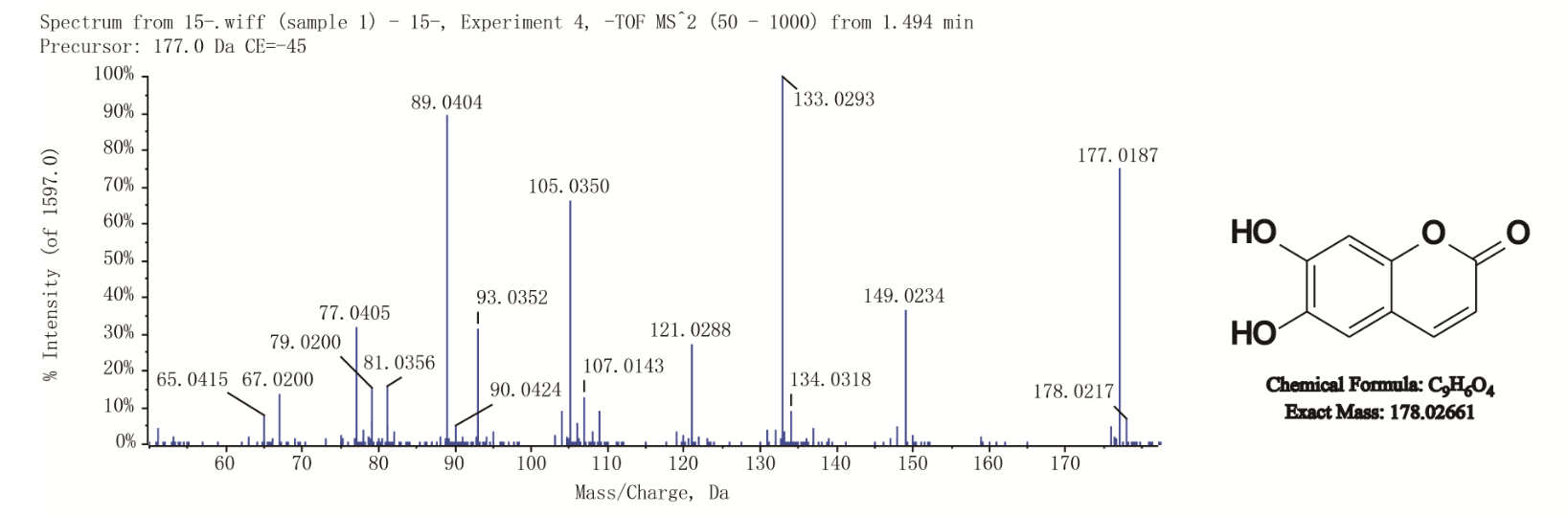


16 Vanillic acid C8H8O4 168.04226


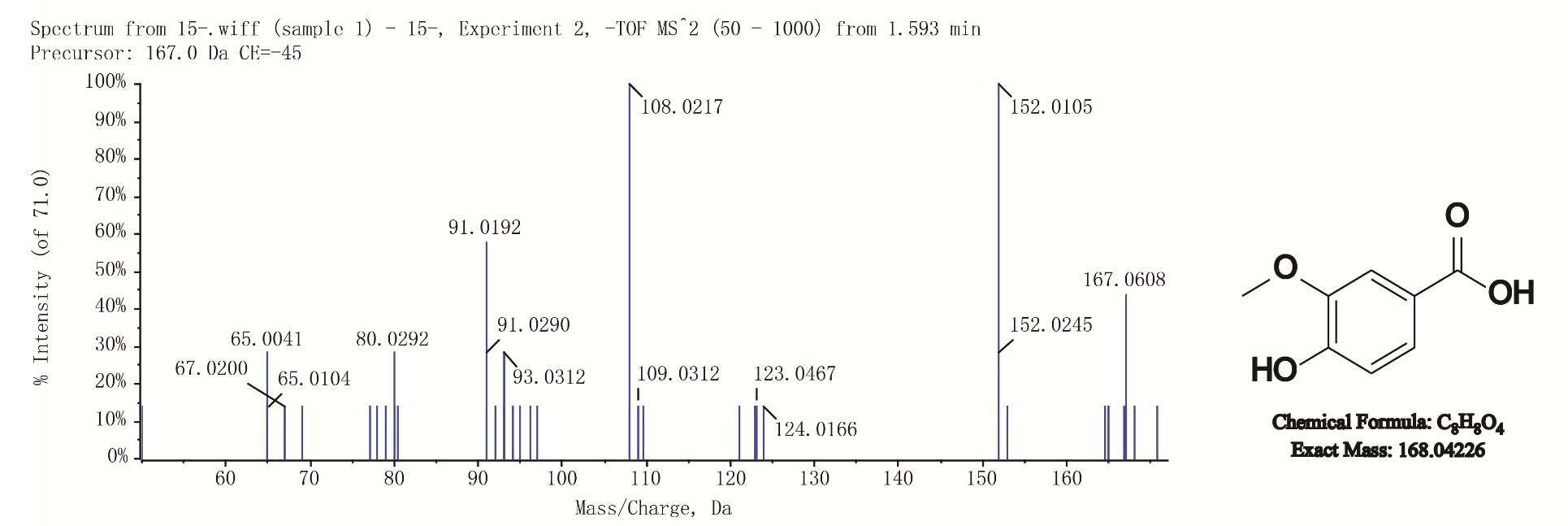


17 4-Hydroxycinnamic acid C9H8O3 164.04734


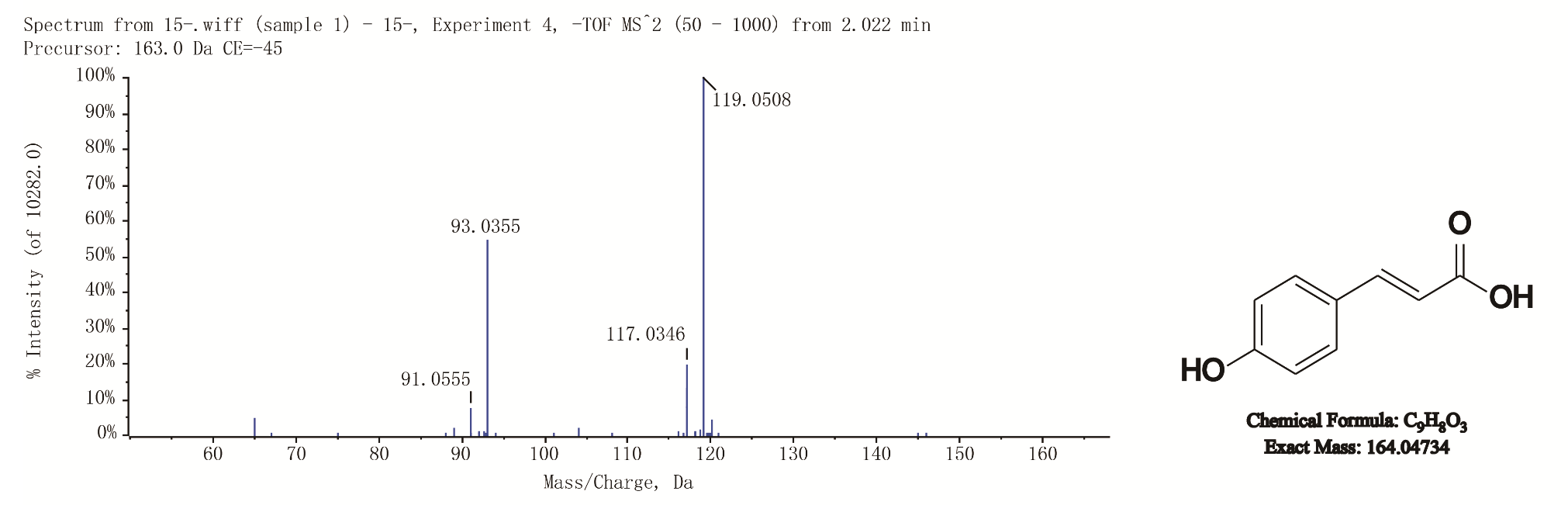


18 Azelaic acid C9H16O4 188.10486


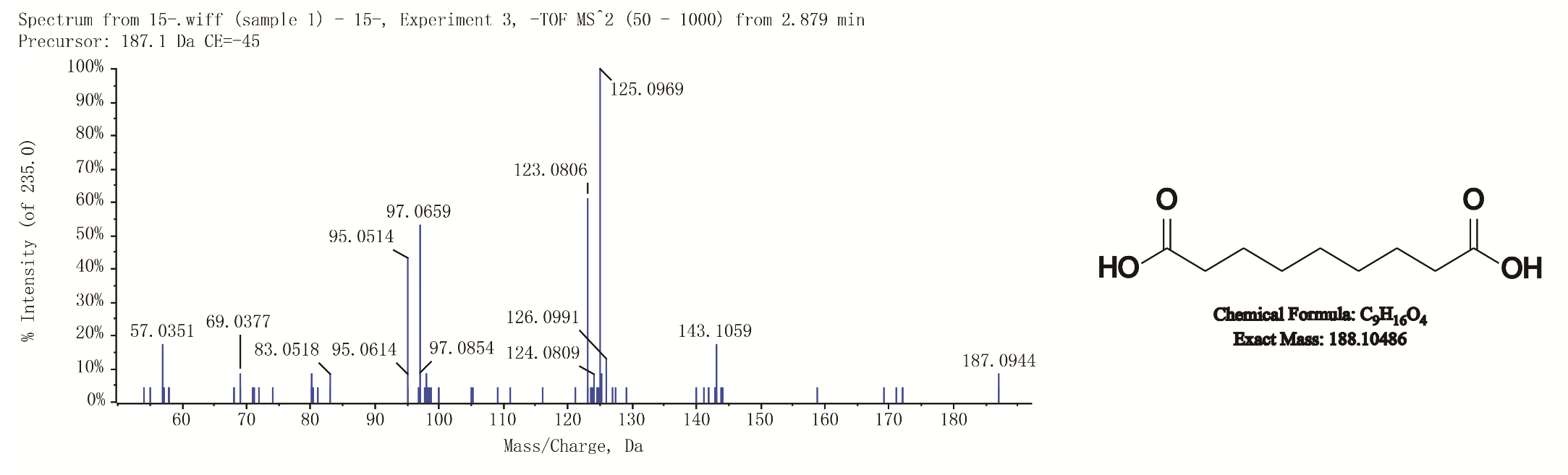


19 Ferulic acid C10H10O4 194.05791


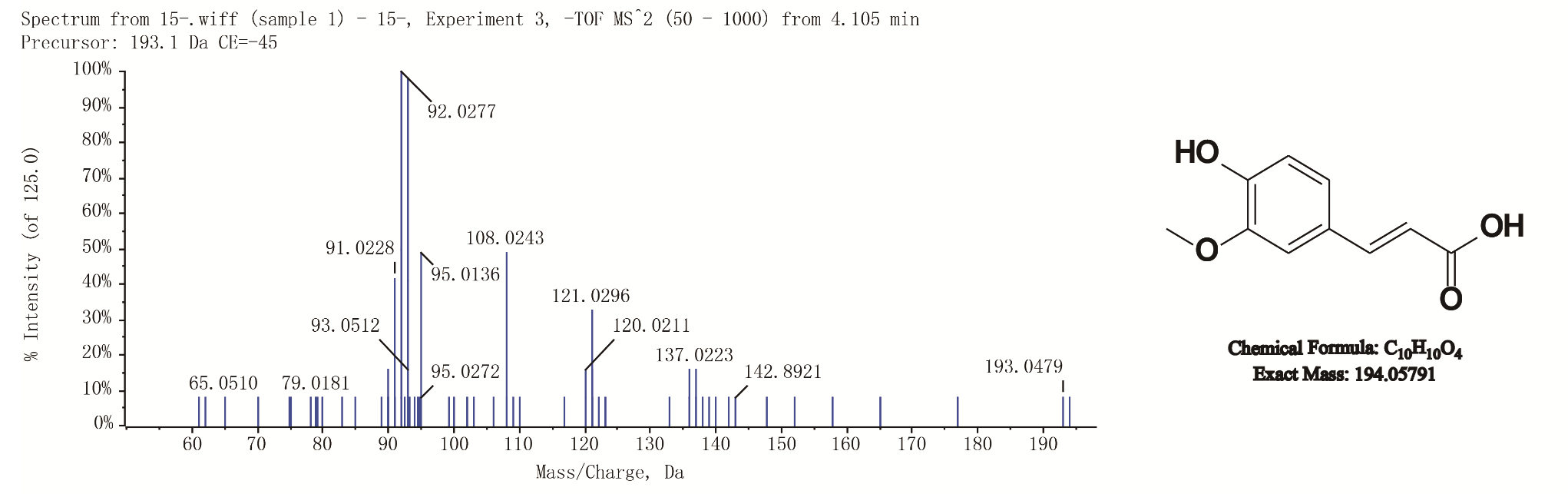


20 Sulfuretin C15H10O5 270.05282


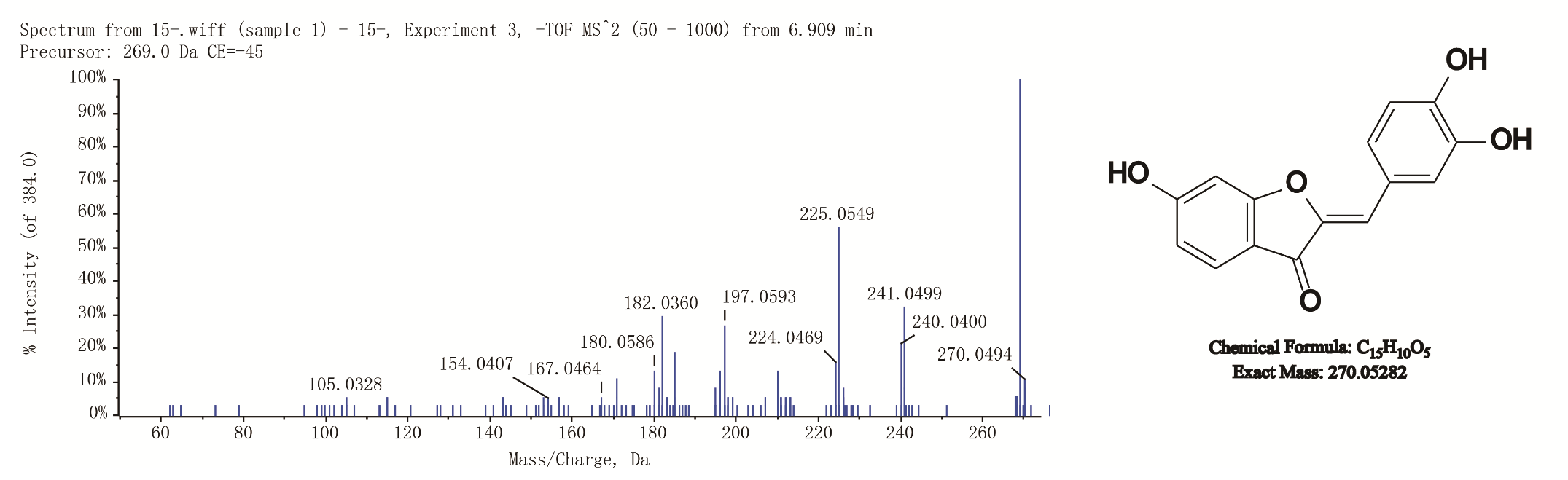


**Supplementary materials (Part C)**

**Acute toxicity test and drug regimen of NFE**

1. **Method**

The dosage design of the NFE was based on an acute toxicity test. A group of 10 Kunming (KM) mice were treated with the maximum concentration of NFE solution for oral administration. The mice were administered three times at 4 h intervals, while the death events and living conditions of mice were observed for 7 days.

1. **Result**

The administered dose of NFE did not elicit any noticeable change in the gain of behavioral disorders or organ toxicity in the acute toxicity test, and no animals died up to the end of the experimental period.

Considering that the clinical dosage of *N. fordii* is 15-40 g per day for adults, and the maximum dosage for mice in acute toxicity test was 2.448 g, the dosing multiple was calculated by the following formula.

$$Dosing multiple= \frac{maximum dose of mice}{average weight of mice}\times\frac{average weight of adults, 60kg}{maximum dose of adults}$$

This showed that the maximum dose of mice was more than 100 times that of the maximum clinical dose in adults. Generally, it indicates very low toxicity of NFE in oral administration. According to a pharmacological methodology (Wei et al., 2010), the dosage of NFE was 1/5, 1/10, and 1/20 of the maximum dose, that is, 400, 200, and 100 mg/kg, respectively.

**Supplementary materials (Part D)**

**The primer sequences of target genes are as follows.**

| Genes | Sequences (5’ to 3’) |
| --- | --- |
| TGF-β Forward | CTTTGTACAACAGCACCCGC |
| TGF-β Reverse | TAGATTGCGTTGTTGCGGTC |
| Smad3 Forward | CAGGAGGAGAAGTGGTGCGA |
| Smad3 Reverse | TCCAGTGACCTGGGGATGGTAA |
| Smad4 Forward | ACATTACTGGCCGGTTCACA |
| Smad4 Reverse | AGGACAGCTTGAAGGGACTT |
| Smad7 Forward | AGGCATTCCTCGGAAGTCAAG |
| Smad7 Reverse | GTCTGGACAGTCTGCAGTTGGTT |
| CTGF Forward | CTTCCCGAGAAGGGTCAAGC |
| CTGF Reverse | TTCCAGTCGGTAGGCAGCTA |
| ERK1/2 Forward | GCTTATCAACACCACCTGCG |
| ERK1/2 Reverse | TTTGGTGTAGCCCTTGGAGTT |
| α-SMA Forward | GGAGATGGCGTGACTCACAA |
| α-SMA Reverse | CGCTCAGCAGTAGTCACGAA |
| β-actin Forward | AGGGAAATCGTGCGTGACAT |
| β-actin Reverse | GAACCGCTCATTGCCGATAG |

# Supplementary Figures and Tables

**Figure S1. Total ion current (TIC) chromatograms of NFE by LC-Q-TOF-MS/MS, including negative ion mode (A) and positive ion mode (B).**


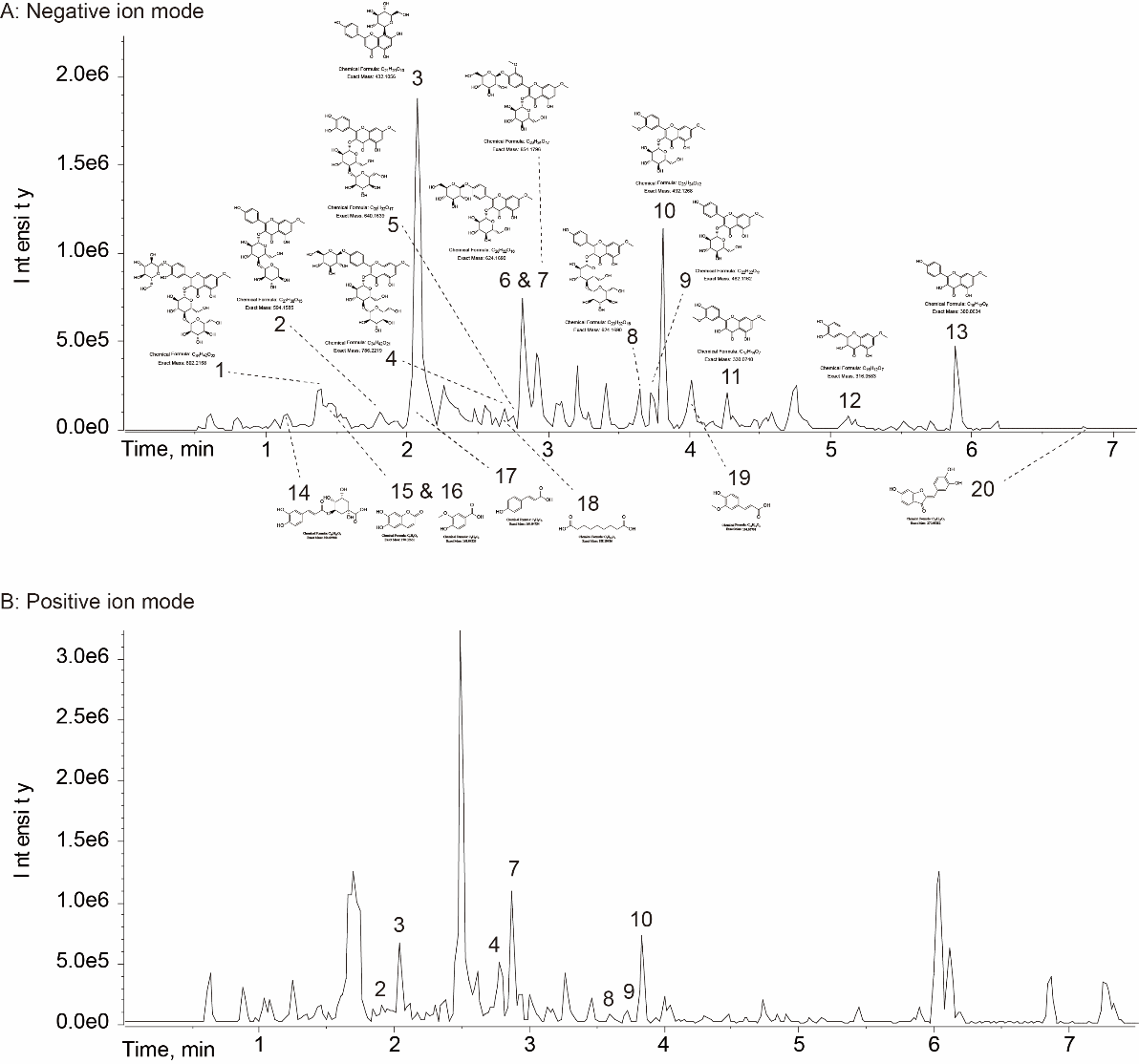


**Figure S2. MRM chromatograms of 5 components.**


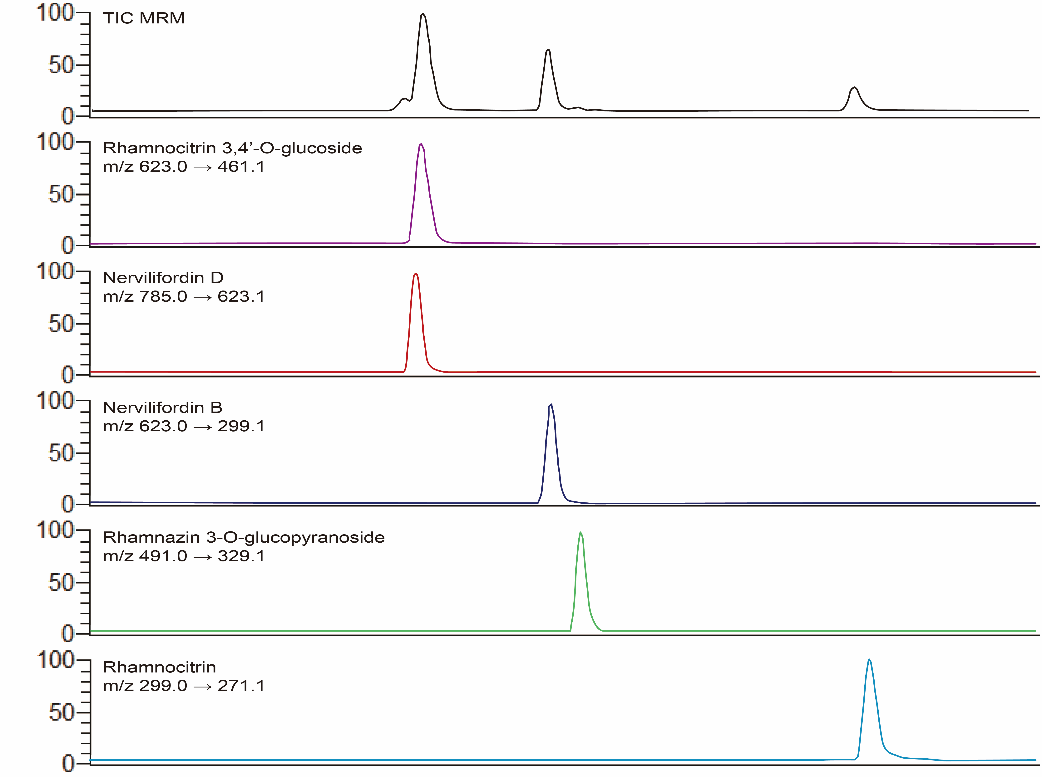


**Table S1(A) Components identified by LC-Q-TOF-MS/MS in NFE (negative ion mode).**

| No | Name | Formular | Mass | **RT** | Adduct | Found at mass | Fragment ions |  | Error |
| --- | --- | --- | --- | --- | --- | --- | --- | --- | --- |
| 1 | Nervilifordin E | C_34_H_42_O_22_ | 802.2168 | **1.40** | -H | 801.2132 | 413.0822 | 293.0453 | 4.6 |
| 2 | Nervilifordin A | C_27_H_30_O_15_ | 594.1585 | **1.81** | -H | 593.1525 | 413.0888 | 293.0456 | 2.2 |
| 3 | Apigenin 8-C-glucoside | C_21_H_20_O_10_ | 432.1057 | **2.03** | -H | 431.0989 | 311.0567 | 283.0607 | 1.1 |
| 4 | Nervilifordin D | C_34_H_42_O_21_ | 786.2219 | **2.73** | +COOH | 831.2240 | 461.1108 | 299.0564 | 6.0 |
| 5 | Nervilifordin C | C_28_H_32_O_17_ | 640.1640 | **2.75** | -H | 639.1587 | 477.1041 | 315.0508 | 3.2 |
| 6 | Rhamnocitrin 3,4'-O-glucoside | C_28_H_32_O_16_ | 624.1690 | **2.82** | +COOH | 669.1696 | 461.1098 | 299.0565 | 5.2 |
| 7 | Rhamnazin 3,4'-O-glucoside | C_29_H_34_O_17_ | 654.1796 | **2.85** | -H | 655.1843 | 491.1207 | 329.0668 | 6.0 |
| 8 | Nervilifordin B | C_28_H_32_O_16_ | 624.1690 | **3.62** | -H | 623.1628 | 299.0559 | 284.0328 | 1.6 |
| 9 | Rhamnocitrin 3-O-glucoside | C_22_H_22_O_11_ | 462.1162 | **3.81** | -H | 461.1094 | 299.0558 | 255.0298 | 0.9 |
| 10 | Rhamnazin 3-O-glucopyranoside | C_23_H_24_O_12_ | 492.1268 | **3.86** | -H | 491.1201 | 329.0664 | 313.0357 | 1.3 |
| 11 | Rhamnazin | C_17_H_14_O_7_ | 330.0740 | **4.19** | -H | 329.0669 | 299.0205 | 271.0270 | 0.5 |
| 12 | Rhamnetin | C_16_H_12_O_7_ | 316.0583 | **5.06** | -H | 315.0518 | 300.0279 | 165.0190 | 2.3 |
| 13 | Rhamnocitrin | C_16_H_12_O_6_ | 300.0634 | **5.89** | -H | 299.0569 | 271.0619 | 255.0307 | 2.6 |
| 14 | Esculetin | C_9_H_6_O_4_ | 178.0266 | **1.08** | -H | 353.0877 | 191.0554 | 173.0454 | -0.3 |
| 15 | Vanillic acid | C_8_H_8_O_4_ | 168.0422 | **1.48** | -H | 177.0194 | 149.0234 | 133.0293 | 0.5 |
| 16 | 4-Hydroxycinnamic acid | C_9_H_8_O_3_ | 164.0473 | **1.57** | -H | 167.0350 | 152.0105 | 108.0217 | 0.3 |
| 17 | Azelaic acid | C_9_H_16_O_4_ | 188.1048 | **2.03** | -H | 163.0400 | 119.0508 | 93.0335 | 2.3 |
| 18 | Ferulic acid | C_10_H_10_O_4_ | 194.0579 | **2.85** | -H | 187.0944 | 143.1059 | 125.0969 | 2.6 |
| 19 | Sulfuretin | C_15_H_10_O_5_ | 270.0528 | **4.07** | -H | 193.0510 | 108.0243 | 92.0277 | 0.3 |
| 20 | Esculetin | C_9_H_6_O_4_ | 178.0266 | **6.91** | -H | 269.0460 | 225.0549 | 197.0593 | 1.9 |

**Table S1(B) Components identified by LC-Q-TOF-MS/MS in NFE (positive ion mode).**

| No | name | formula | mass | **RT** | adduct | found at mass | Fragment ion |  | error |
| --- | --- | --- | --- | --- | --- | --- | --- | --- | --- |
| 1 | Nervilifordin E | C_34_H_42_O_22_ | 802.2168 |  |  |  |  |  |  |
| 2 | Nervilifordin A | C_27_H_30_O_15_ | 594.1585 | **1.91** | +H | 595.1643 | 415.1029 | 313.0710 | 2.50 |
| 3 | Apigenin 8-C-glucoside | C_21_H_20_O_10_ | 432.1057 | **2.06** | +H | 433.1112 | 313.0703 | 283.0596 | 3.97 |
| 4 | Nervilifordin D | C_34_H_42_O_21_ | 786.2219 | **2.78** | +H | 787.2261 | 463.1225 | 301.0705 | 3.81 |
| 8 | Nervilifordin B | C_28_H_32_O_16_ | 624.1690 | **3.67** | +H | 625.1738 | 463.1200 | 284.0465 | 3.97 |
| 9 | Rhamnocitrin 3-O-glucoside | C_22_H_22_O_11_ | 462.1162 | **3.84** | +H | 463.1218 | 286.0469 | 167.0341 | 3.54 |
| 10 | Rhamnazin 3-O-glucopyranoside | C_23_H_24_O_12_ | 492.1268 | **3.88** | +H | 493.1323 | 331.0804 | 316.0586 | 3.60 |

**Table S2 Linear-regression, LODs, and LOQs of the five components**

| Components | Regression Equation  ng/mL | Linear Range  ng/ml | Correlation  Coefficient  R^2 | LOD  pg/ml | LOQ  pg/ml |
| --- | --- | --- | --- | --- | --- |
| **1** Rhamnocitrin 3,4’-O-glucoside | $\hat{y}$ = 1942.4X+4401.6 | 2000~40000 | 0.9990 | 658.8 | 2173.9 |
| **2** Nervilifordin D | $\hat{y}$ = 724.86X+50.313 | 500~10000 | 0.9992 | 7692.3 | 23076.9 |
| **3** Nervilifordin B | $\hat{y}$ = 19964X+87.362 | 100~2000 | 0.9997 | 333.3 | 1110.9 |
| **4** Rhamnazin 3-O-glucopyranoside | $\hat{y}$ = 17679X-0.77 | 5~100 | 0.9991 | 21.3 | 70.9 |
| **5** Rhamnocitrin | $\hat{y}$ = 128772X-154.68 | 10~200 | 0.9994 | 30.6 | 102.0 |

**Table S3 Precision, repeatability, and stability of the five components**

| Components | Precision  Intra-Day  RSD /% | Precision  Inter-Day  RSD /% | Repeatability  RSD /% | Stability  RSD /% |
| --- | --- | --- | --- | --- |
| **1** Rhamnocitrin 3,4’-O-glucoside | 2.74 | 2.83 | 2.92 | 2.55 |
| **2** Nervilifordin D | 2.68 | 2.97 | 2.50 | 2.87 |
| **3** Nervilifordin B | 2.70 | 2.79 | 2.79 | 2.43 |
| **4** Rhamnazin 3-O-glucopyranoside | 2.62 | 2.90 | 2.21 | 2.27 |
| **5** Rhamnocitrin | 1.55 | 1.69 | 2.60 | 1.51 |

**Table S4 Coefficient of recovery of the five components**

| Components | Initial  Amount  /μg | Added  Amount  /μg | Detected  Amount  /μg | Recovery  /% | Average  Recovery  /% | RSD  /% |
| --- | --- | --- | --- | --- | --- | --- |
| **1** Rhamnocitrin 3,4'-O-glucoside | 407.83 | 500.00 | 927.04 | 103.8 | 100.8 | 2.71 |
|  | 406.61 | 500.00 | 921.69 | 103.2 |  |  |
|  | 409.86 | 500.00 | 923.43 | 102.7 |  |  |
|  | 404.58 | 500.00 | 897.48 | 98.6 |  |  |
|  | 409.86 | 500.00 | 895.69 | 97.2 |  |  |
|  | 408.23 | 500.00 | 906.20 | 99.6 |  |  |
| **2** Nervilifordin D | 128.6 | 140.00 | 271.28 | 102.0 | 100.2 | 2.32 |
|  | 128.55 | 140.00 | 270.47 | 101.6 |  |  |
|  | 129.17 | 140.00 | 270.62 | 101.0 |  |  |
|  | 127.53 | 140.00 | 264.89 | 98.1 |  |  |
|  | 129.19 | 140.00 | 271.56 | 102.1 |  |  |
|  | 128.68 | 140.00 | 264.35 | 96.5 |  |  |
| **3** Nervilifordin B | 10.04 | 7.52 | 8.50 | 16.34 | 101.5 | 2.67 |
|  | 10.01 | 7.50 | 8.50 | 16.28 |  |  |
|  | 10.09 | 7.56 | 8.50 | 16.39 |  |  |
|  | 9.96 | 7.46 | 8.50 | 16.12 |  |  |
|  | 10.09 | 7.56 | 8.50 | 15.89 |  |  |
|  | 10.05 | 7.53 | 8.50 | 15.88 |  |  |
| **4** Rhamnazin 3-O-glucopyranoside | 2.05 | 1.80 | 3.89 | 102.5 | 98.9 | 2.78 |
|  | 2.04 | 1.80 | 3.73 | 94.0 |  |  |
|  | 2.06 | 1.80 | 3.85 | 99.5 |  |  |
|  | 2.03 | 1.80 | 3.81 | 98.9 |  |  |
|  | 2.06 | 1.80 | 3.84 | 99.1 |  |  |
|  | 2.05 | 1.80 | 3.84 | 99.5 |  |  |
| **5** Rhamnocitrin | 3.85 | 2.50 | 6.44 | 103.3 | 100.1 | 2.39 |
|  | 3.84 | 2.50 | 6.31 | 98.6 |  |  |
|  | 3.87 | 2.50 | 6.35 | 99.1 |  |  |
|  | 3.82 | 2.50 | 6.28 | 98.3 |  |  |
|  | 8.88 | 2.50 | 6.33 | 98.4 |  |  |
|  | 3.86 | 2.50 | 6.44 | 103.2 |  |  |

**Table S5 Retention time, monitoring ion pairs, and UHPLC-MS data of five components**

| Components | Molecular Formula | t_R_/min | Parent Mass  (m/z) | Product Mass  (m/z) | CE  /eV | Ion  Mode |
| --- | --- | --- | --- | --- | --- | --- |
| **1** Rhamnocitrin 3,4’-O-glucoside | 624 | 2.8 | 623.0 | 461.1 | 5 | Negative |
| **2** Nervilifordin D | 786 | 2.7 | 785.0 | 623.1 | 6 | Negative |
| **3** Nervilifordin B | 624 | 3.5 | 623.0 | 299.1 | 45 | Negative |
| **4** Rhamnazin 3-O-glucopyranoside | 492 | 3.7 | 491.0 | 329.1 | 33 | Negative |
| **5** Rhamnocitrin | 300 | 5.1 | 299.0 | 271.1 | 9 | Negative |

**Reference**

Wei, W., Ximei, W., and Yuanjian, L. (2010). *Experimental Methodology of Pharmacology.* Beijing: People's Medical Publishing House. ISBN 978-7-117-12478-2.

Zhang, L., Zhao, Z., Lin, C., and Zhu, C. (2012a). Studies on Chemical Constituents from Whole Plants of Nervilia fordii (Hance) Schltr. *Traditional Chinese Drug Research & Clinical Pharmacology* 23**,** 453-455.

Zhang, L., Zhao, Z.X., Lin, C.Z., Zhu, C.C., and Gao, L. (2012b). Three new flavonol glycosides from Nervilia fordii. *Phytochemistry Letters* 5**,** 104-107.
